# Supplementary material for: The arbitrium system controls prophage induction
Source: Curr Biol. 2021 Nov 22;31(22):5037–5045.e3. doi: 10.1016/j.cub.2021.08.072 (PMC8612738; doi:10.1016/j.cub.2021.08.072)
Supplement: Document S1. Figures S1–S7 and Tables S1–S5 [file mmc1.pdf]

Current Biology, Volume *31*

Supplemental Information

The arbitrium system controls prophage induction

Aisling Brady, Nuria Quiles-Puchalt, Francisca Gallego del Sol, Sara Zamora-Caballero, Alonso Felipe-Ruíz, Jorge Val-Calvo, Wilfried J.J. Meijer, Alberto Marina, and José R. Penadés

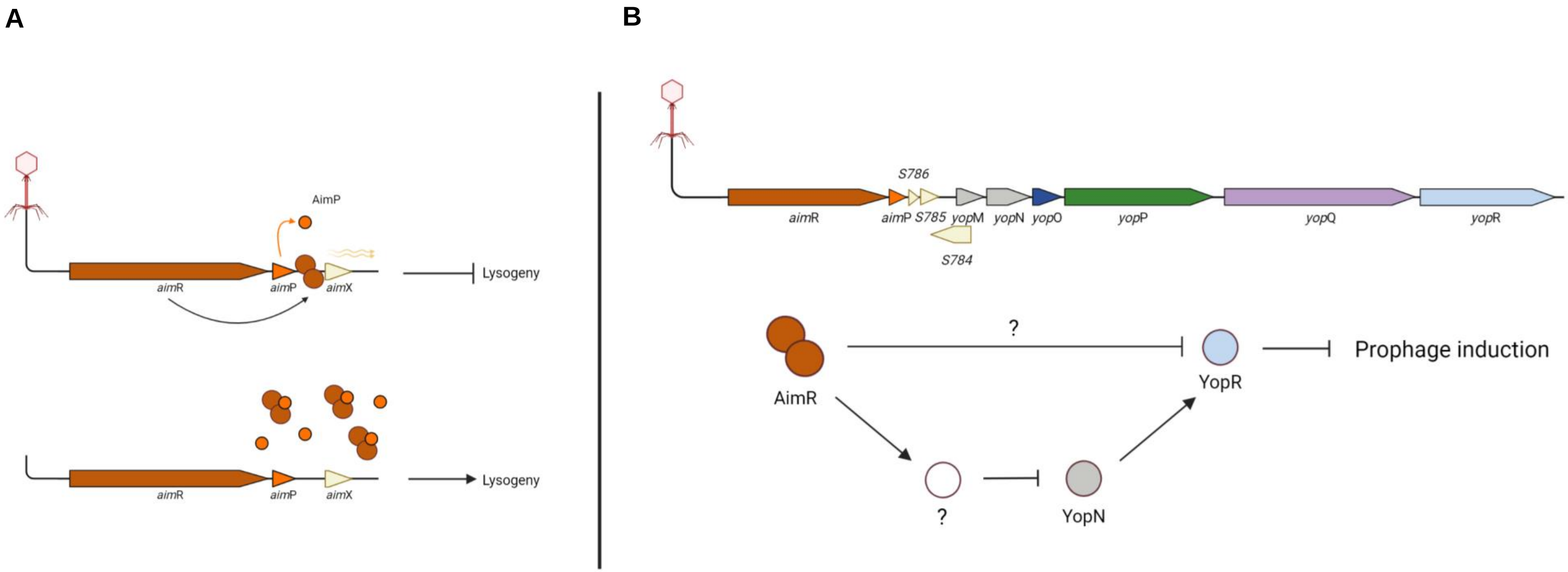

Figure S1. Model for the mechanism of action of the arbitrium system in phages of the SPβ family, Related to Figure 3. (A) The arbitrium model prior to this study describes that after infection of a SPβ prophage, AimR is being expressed and binds to the operator site promoting expression of the *aim*X sRNA and promoting the lytic cycle. After AimP accumulates above the threshold levels, it binds to AimR disrupting its binding to the DNA and reducing expression of *aim*X, leading to lysogeny. (B) Our understanding is that the arbitrium system of SPβ is involved in a more complex mechanism to control prophage induction. The regulator AimR functions to inhibit the phage repressor, YopR, thus promoting prophage induction. Another component of the system is YopN that we hypothesise to promote YopR activity, acting as a negative regulator of prophage induction. We propose that following activation of the SOS response, AimR activates an unknown component that blocks YopN function, thus reducing the activity of YopR and promoting induction.

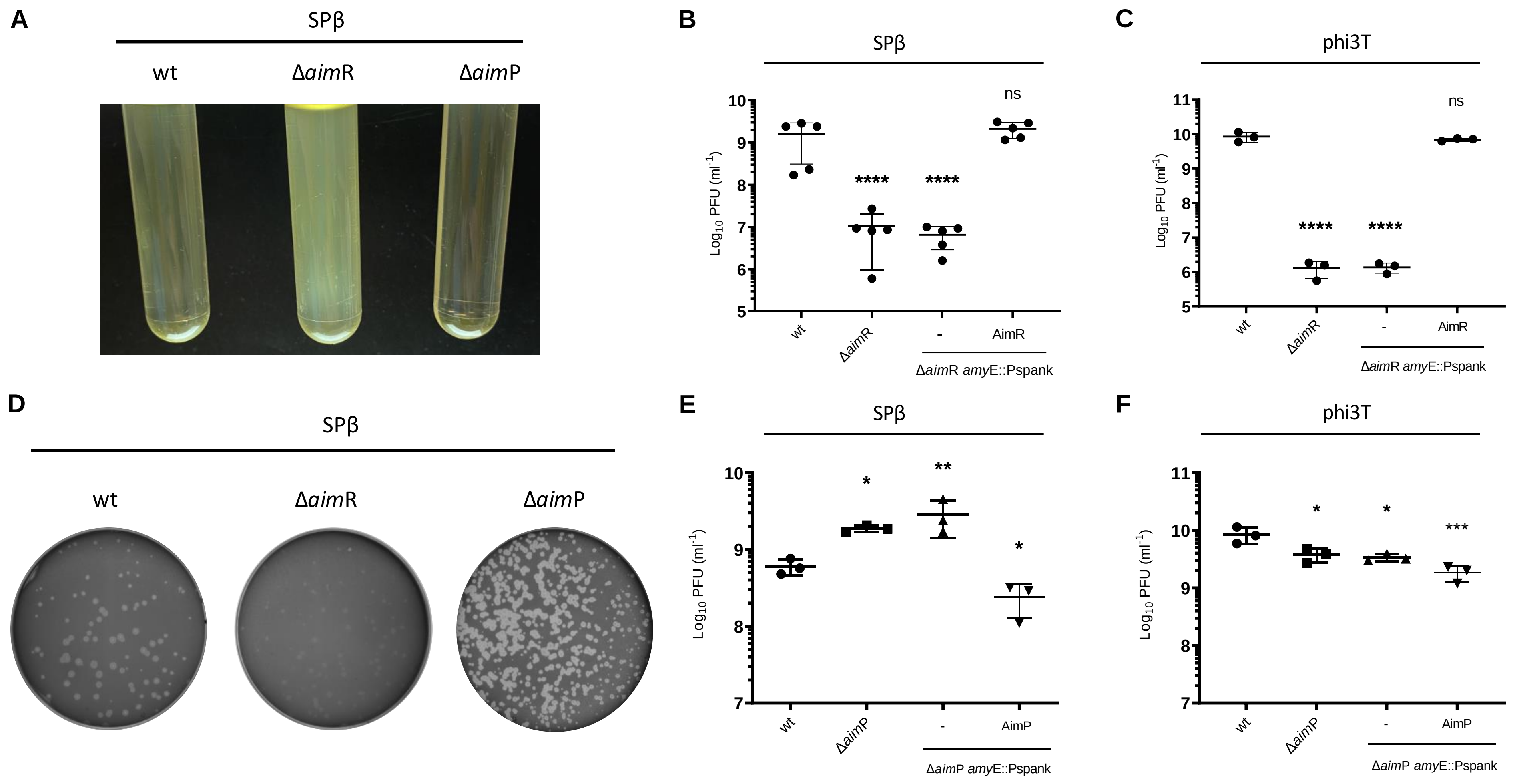

Figure S2. Analysis of the *aim*R and *aim*P deletions in SPβ-type prophages, Related to Figure 5. (A) Lysogenic strains for phage SPβ, SPβ Δ*aim*R or SPβ Δ*aim*P were MC induced (0.5 μg/ml) and incubated at 30 °C with 80 rpm shaking for 4 h. The lysates were left overnight at room temperature before being photographed. (B) Complementation of the *aim*R mutant in SPβ. Strains lysogenic for phage SPβ wt, Δ*aim*R, Δ*aim*R *amy*E::Pspank and Δ*aim*R *amy*E::Pspank-AimR were MC induced (0.5 μg/ml) and the number of resulting phages were quantified by titering using 168 Δ6 as the recipient strain. The results are represented as the plaque forming units (PFUs) mL^{-1}. The means and SDs are presented (n = 5). An ordinary one-way ANOVA of transformed data was performed to compare mean differences between titres. Adjusted p values were as follows: SPβ Δ*aim*R ****p = < 0.0001; Δ*aim*R *amy*E::Pspank ****p = < 0.0001; Δ*aim*R *amy*E::Pspank-AimR ns = not significant. (C) Complementation of the *aim*R mutant in phi3T. Strains lysogenic for phages phi3T wt, Δ*aim*R, Δ*aim*R *amy*E::Pspank and Δ*aim*R *amy*E::Pspank-AimR were MC induced (0.5 μg/ml) and the number of resulting phages were quantified by titering using 168 Δ6 as the recipient strain. The results are represented as PFUs/mL^{-1}. The means and SDs are presented (n = 3). An ordinary one-way ANOVA of transformed data was performed to compare mean differences between titres. Adjusted p values were as follows: SPβ Δ*aim*R ****p = < 0.0001; Δ*aim*R *amy*E::Pspank ****p = < 0.0001; Δ*aim*R *amy*E::Pspank-AimR ns = not significant. (D) SPβ lysates were tittered using 168 Δ6 as the recipient strain. The resulting plaque morphologies were photographed. (E) Strains lysogenic for phage SPβ wt, Δ*aim*P, Δ*aim*P *amy*E::Pspank and Δ*aim*P *amy*E::Pspank-AimP were MC induced (0.5 μg/ml) and the number of resulting phages were quantified by titering using 168 Δ6 as the recipient strain. The results are represented as the plaque forming units (PFUs) mL^{-1}. The means and SDs are presented (n = 3). An ordinary one-way ANOVA of transformed data was performed to compare mean differences between titres. Adjusted p values were as follows: SPβ Δ*aim*P *p = 0.0205; Δ*aim*P *amy*E::Pspank **p = 0.0049; Δ*aim*P *amy*E::Pspank-AimP *p = 0.0391. (F) Strains lysogenic for phages phi3T wt, Δ*aim*P, Δ*aim*P *amy*E::Pspank and Δ*aim*P *amy*E::Pspank-AimP were MC induced (0.5 μg/ml) and the number of resulting phages were quantified by titering using 168 Δ6 as the recipient strain. The results are represented as PFUs/mL^{-1}. The means and SDs are presented (n = 3). An ordinary one-way ANOVA of transformed data was performed to compare mean differences between titres. Adjusted p values were as follows: phi3T Δ*aim*P *p = 0.0220; Δ*aim*P *amy*E::Pspank *p = 0.0125; Δ*aim*P *amy*E::Pspank-AimP ***p = 0.0005.

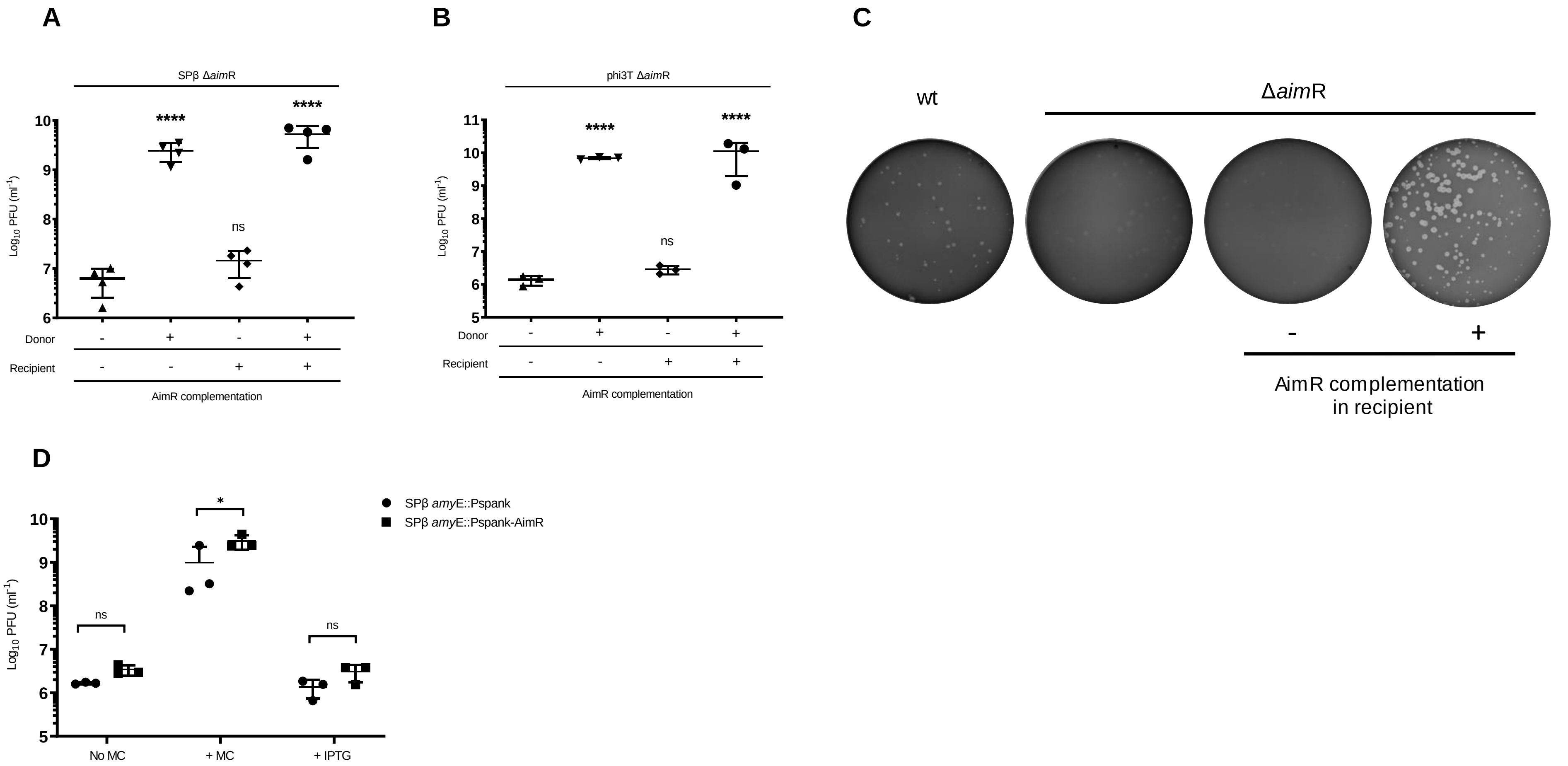

Figure S3. Complementation of the *aim*R mutants in recipient strain , Related to Figure 4 and Figure 5. (A) Strain lysogenic for phage SPβ Δ*aim*R was MC induced (0.5 µg/ml) and the number of resulting phages were quantified by titering using 168 Δ6 *amy*E::Pspank (-) or 168 Δ6 *amy*E::Pspank-AimR$_{SPβ}$ (+) as recipient strains. The results are represented as the plaque forming units (PFUs) mL^{-1}. The means and SDs are presented (n = 4). An ordinary one-way ANOVA of transformed data was performed to compare mean differences between titres. Adjusted p values were as follows: column A vs column B ****p = <0.0001; column A vs column C ns = not significant; column A vs column D ****p = <0.0001. (B) Strain lysogenic for phage phi3T Δ*aim*R was MC induced (0.5 µg/ml) and the number of resulting phages were quantified by titering using 168 Δ6 *amy*E::Pspank (-) or 168 Δ6 and Δ*aim*R *amy*E::Pspank-AimR$_{phi3T}$ (+) as recipient strains. The results are represented as the plaque forming units (PFUs) mL^{-1}. The means and SDs are presented (n = 3). An ordinary one-way ANOVA of transformed data was performed to compare mean differences between titres. . Adjusted p values were as follows: column A vs column B ****p = <0.0001; column A vs column C ns = not significant; column A vs column D ****p = <0.0001. (C) Plaques morphologies produced after titration of the SPβ Δ*aim*R using 168 Δ6 *amy*E::Pspank (-) or 168 Δ6 *amy*E::Pspank-AimR$_{SPβ}$ (+) as recipient strains were photographed. (D) Overexpression of AimR does not induce the lytic cycle. Strains lysogenic for phage SPβ *amy*E::Pspank and SPβ *amy*E::Pspank-AimR$_{SPβ}$ were analysed for their ability to produce phage particles under several conditions: without induction (No MC), with phage induction (+ MC 0.5 µg/ml) and with Pspank induction (+ IPTG 1mM). The number of resulting phages were quantified by titering using 168 Δ6 as the recipient strain. The results are represented as the plaque forming units (PFUs) mL^{-1}. The means and SDs are presented (n = 3). An ordinary one-way ANOVA of transformed data was performed to compare mean differences between titres. Differences in titer with “No MC” and “+ IPTG” were not significant (ns). The adjusted p value comparing SPβ *amy*E::Pspank and *amy*E::Pspank-AimR + MC *p = 0.0227.

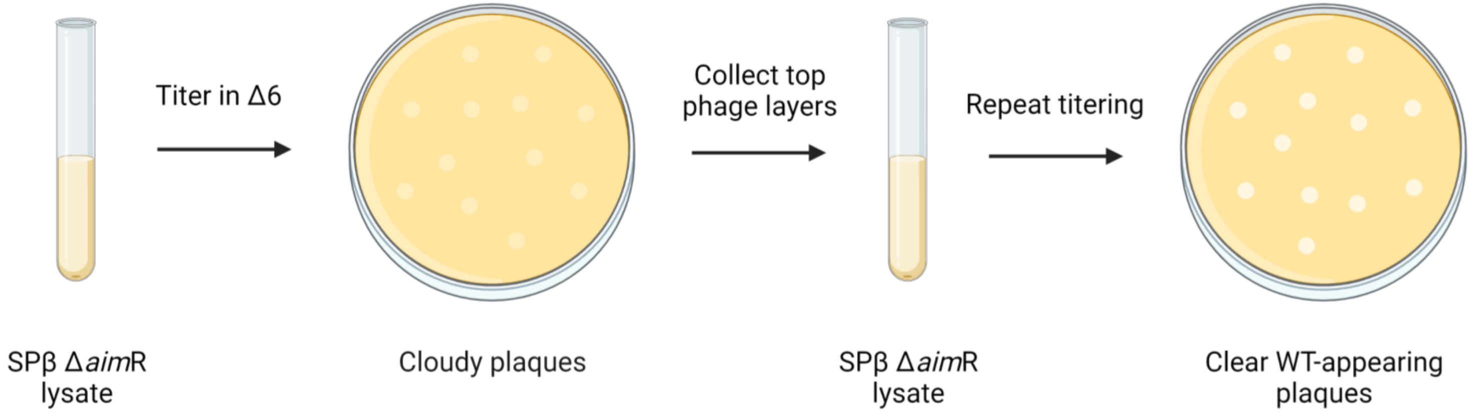

Figure S4. Schematic representation of the SPβ Δ*aim*R evolution procedure Related to STAR Methods. SPβ Δ*aim*R lysate was acquired following MC induction of a lysogenic strain carrying the SPβ Δ*aim*R phage. The lysate was titered using 168 Δ6 as the recipient strain and the resulting cloudy plaques were collected and passaged, as described in the STAR Methods, until wt-appearing plaques were obtained. Created with BioRender.com

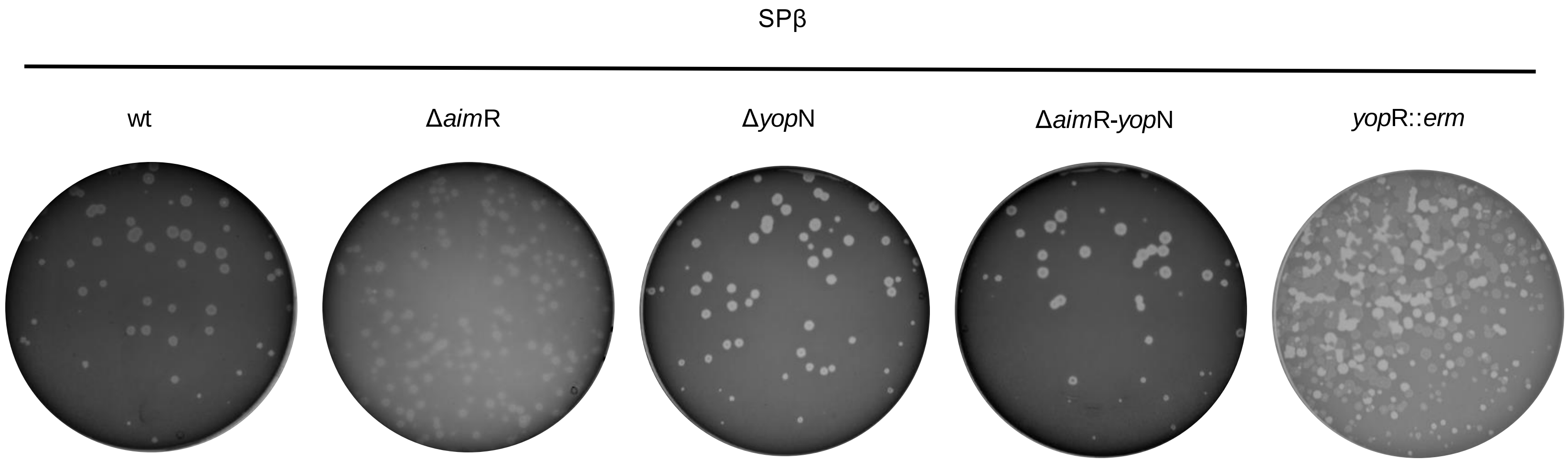

Figure S5. Plaque morphology of SPβ wt, Δ*aim*R, Δ*yop*N, Δ*aim*R-*yop*N and *yop*R::*erm* phages, Related to Figure 5 and Figure 6. Strains lysogenic for phage SPβ wt, Δ*aim*R, Δ*yop*N and Δ*aim*R-*yop*N were MC induced (0.5 μg/ml) and titered using 168 Δ6 as the recipient strain. A strain lysogenic for phage SPβ was transformed with an erythromycin cassette to replace the *yop*R gene. The resulting strain, supposedly *yop*R::*erm*, was MC induced (0.5 μg/ml) and titered using 168 Δ6 as the recipient strain. The resulting plaque morphologies were photographed.

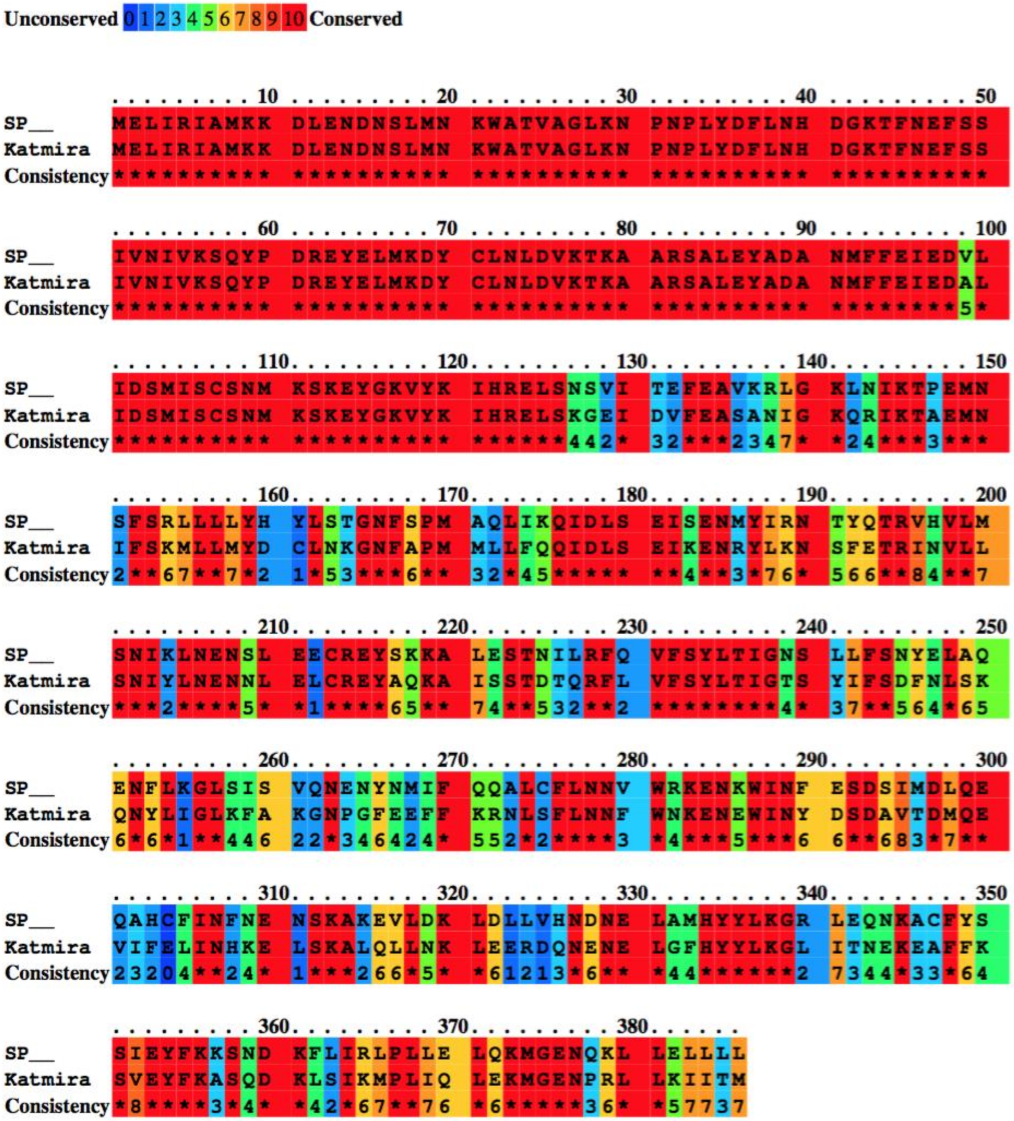

Figure S6. Homology analysis of AimR$_{SP\beta}$ and AimR$_{KATMIRA1933}$, Related to STAR Methods. AimR sequences from SPβ and KATMIRA1933 were obtained from BLAST. The superposition analysis was made using the PRALINE program. Residues conservancy is depicted by blue to red colours.

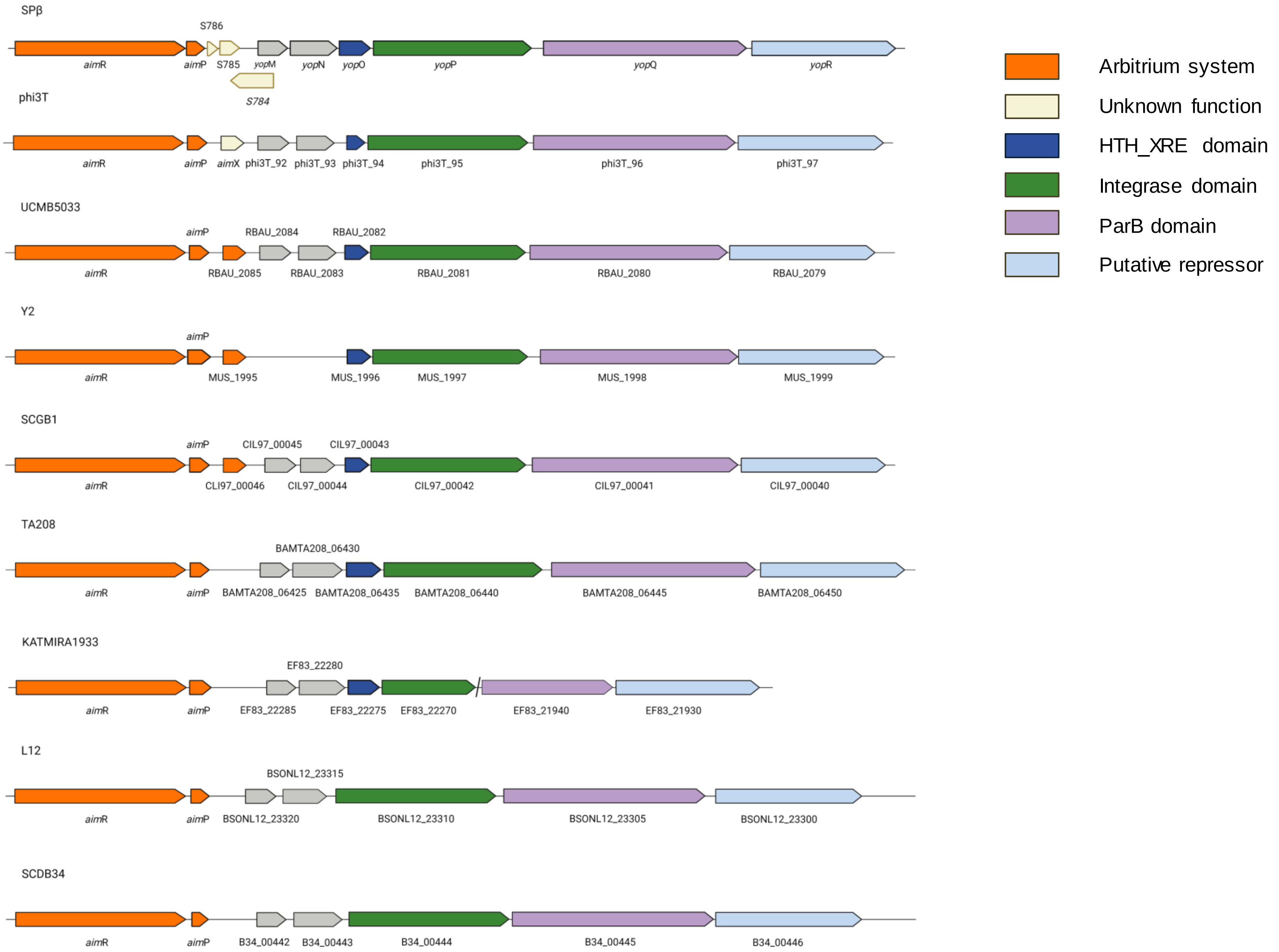

Figure S7. Schematic representation of the SPβ-like phages arbitrium and operon genetic layout, Related to Figure 3. Diagram shows the genetic organisation of the arbitrium genes, *aim*R and *aim*P, followed by the operon directly downstream. Colours denote putative functions according to BLAST results; orange: arbitrium genes, grey: unknown function, navy blue: HTH_XRE domain, green: integrase domain, purple: ParB domain, light blue: putative repressor. Rotated black line indicates the end/beginning of the contigs containing the genes described for Katmira1933. Created with BioRender.com

Strain	Gene	Mutation
JP20762	*yop*N	L90S
JP20766	*yop*N	L46P
	*yop*Q	T156T
JP20769	*yop*N	I51* Deletion produces frameshift and stop codon
Lytic phage 1	*yop*R	L140* Deletion produces frameshift and stop codon
Lytic phage 2	*yop*R	L49* Deletion produces frameshift and stop codon

Table S1. Mutations identified in evolved SPβ Δ*aim*R phages, Related to Figure 3 and Figure 4.

Phage/lysogen	AimR	AimP	AimP sequence	Operon genes accession numbers					
				Gene 1	Gene 2	Gene 3	Gene 4	Gene 5	Gene 6
SPβ	GenBank: NP_389968	GenBank: NP_389967	GMPRGA	GenBank: NP_389966	GenBank: NP_389965	GenBank: NP_389964	GenBank: NP_389963	GenBank: NP_389962	GenBank: NP_389961
phi3T	GenBank: APD21232	GenBank: APD21233	SAIRGA	GenBank: APD21235	GenBank: APD21236	GenBank: APD21237	GenBank: APD21238	GenBank: APD21239	GenBank: APD21240
Bacillus amyloliquefaciens UCMB5033	GenBank: CDG30054	*NA	SPSRGA	GenBank: CDG30052	GenBank: CDG30051	GenBank: CDG30050	GenBank: CDG30049	GenBank: CDG30048	GenBank: CDG30047
Bacillus velezensis strain SCGB 1	GenBank: ATC49385	GenBank: ATC49384	SIIRGA	GenBank: ATC49382	GenBank: ATC49381	GenBank: ATC49380	GenBank: ATC49379	GenBank: ATC49378	GenBank: ATC49377
Bacillus amyloliquefaciens TA208	GenBank: AEB23458	GenBank: AEB23459	GVVRGA	GenBank: AEB23460	GenBank: AEB23461	GenBank: AEB23462	GenBank: AEB23463	GenBank: AEB23464	GenBank: AEB23465
Bacillus atrophaeus BA59	GenBank: ATO28982	GenBank: ATO28981	GMPRGA	GenBank: ATO28980	*NA	GenBank: ATO28979	*NA	GenBank: ATO28978	GenBank: ATO28977
Bacillus subtilis KATMIRA1933	GenBank: WP_033885437	GenBank: WP_134819006	GIVRGA	GenBank: WP_033885435	GenBank: WP_009967507	GenBank: WP_019712296	GenBank: WP_033885434	GenBank: NP_389962.1	GenBank: WP_003231032
Bacillus sonorensis L12	GenBank: WP_051056713	GenBank: WP_141231111	GFPRGA	GenBank: WP_006640569	GenBank: WP_006640568	*NA	GenBank: WP_006640567	GenBank: WP_006640566	GenBank: WP_006640565
Bacillus licheniformis strain SCDB 34	GenBank: ARC67883	GenBank: ARC67884	GFTVGA	GenBank: ARC67885	GenBank: ARC67886	*NA	GenBank: ARC67887	GenBank: ARC67888	GenBank: ARC67889

*NA: Not annotated

Table S2. Genetic composition of the arbitrium-operon region in the different SPβ-like phage families, Related to Figure 3.

Strains	Genotype or description	Reference or source
Bacillus subtilis		
168 (1A700)	*trpC2*	S1
Δ6 (1A1299)	*trpC2*; ΔSPβ ; subclacin 168-sensitive; Δ*skin*; ΔPBSX; Δprophage 1; Δ*pks*::Cm; Δprophage 3; Cm^r	S2
IL26	phi3T	S3
BKK20860	*trpC2* Δ*aim*R::*kan*	S4
BKE20860	*trpC2* Δ*aim*R::*erm*	S4
BKE20850	*trpC2* Δ*aim*P::*erm*	S4
BKE20830	*trpC2* Δ*yop*N::*erm*	S4
BKE20790	*trpC2* Δ*yop*R::*erm*	S4
JP22770	*trpC2 SP*β Δ*aim*R	This study
JP22771	*trpC2 SP*β Δ*aim*P	This study
JP22776	*trpC2 SPβ ΔaimR*; *amy*E::P_{spank}	This study
JP22777	trpC2 SPβ ΔaimR; *amy*E::P_{spank}*aim*$R_{SPβ}$	This study
JP19877	Δ6 lysogenic SPβ	This study
JP19936	Δ6 lysogenic SPβ Δ*aim*R	This study
JP20866	Δ6 lysogenic SPβ *yok*I::*kan*	This study
JP22949	Δ6 lysogenic SPβ *yok*I::*kan* Δ*aim*R	This study
JP21702	Δ6 lysogenic SPβ *yok*I::*kan* Δ*aim*P	This study
JP22950	Δ6 lysogenic SPβ *yok*I::kan Δ*aim*R; *amy*E::P_{spank}	This study
JP22951	Δ6 lysogenic SPβ *yok*I::kan Δ*aim*R; *amy*E::P_{spank}*aim*$R_{SPβ}$	This study
JP21854	Δ6 lysogenic phi3T	This study
JP21870	Δ6 lysogenic phi3T *phi3T_5*::*kan*	This study
JP22453	Δ6 lysogenic phi3T *phi3T_5*::*kan* Δ*aim*R	This study
JP22454	Δ6 lysogenic phi3T *phi3T_5*::*kan* Δ*aim*P	This study
JP22518	Δ6 lysogenic phi3T *phi3T_5*::*kan* Δ*aim*R; *amy*E::P_{spank}	This study
JP22519	Δ6 lysogenic phi3T *phi3T_5*::*kan* Δ*aim*R; *amy*E::P_{spank}*aim*$R_{SPβ}$	This study
JP20762	Δ6 lysogenic SPβ Δ*aim*R; *yop*N L90S	This study
JP20766	Δ6 lysogenic SPβ *yok*I::*kan* Δ*aim*R; *yop*N L46P; *yop*Q T156T	This study
JP20769	Δ6 lysogenic SPβ *yok*I::*kan* Δ*aim*R; *yop*N A49*	This study
JP22952	Δ6 lysogenic SPβ Δ*yop*N	This study
JP22953	Δ6 lysogenic SPβ Δ*aim*R Δ*yop*N	This study
JP21752	Δ6 lysogenic SPβ *yop*R::*erm*	This study
JP22339	Δ6 lysogenic SPβ *yop*R::*erm; amy*E::P_{spank}*yop*$R_{SPβ}$	This study
JP19679	Δ6 *amy*E::P_{spank}	This study
JP19944	Δ6 *amy*E::P_{spank}*aim*$R_{SPβ}$	This study
JP22515	Δ6 *amy*E::P_{spank}*aim*R_{3T}	This study
JP21941	Δ6 *amy*E::P_{spank}*yop*$R_{SPβ}$	This study
JP19883	Δ6 lysogenic SPβ; *amy*E::P_{spank}	This study

Table S3. Bacterial strains, Related to STAR Methods.

Plasmid	Description	Reference or source
pDR244	*B. subtilis* thermosensitive vector containing Cre recombinase that allows excision of DNA fragments flanked by *lox*P sites	S4
pDR110	*B. subtilis amy*E integration vector containing IPTG-inducible P_{spank} promoter	S5
pJP2340	*aim*$R_{SP\beta}$ gene cloned in integration vector pDR110	This study
pJP2801	*aim*R_{3T} gene cloned in integration vector pDR111	This study
pJP2800	*yop*$R_{SP\beta}$ gene cloned in integration vector pDR110	This study

Table S4. Plasmids used in this study, Related to STAR Methods.

Mutants		Oligonucleotides	Sequence (5'-3')
kan marker without *lox*P		KanR-5m	TTTGATTTTTAATGGATAATGTGATATAATC
		KanR-6c	TCTAGGTACTAAAACAATTCATCC
erm marker with *lox*P		ErmR-1m	CAGGCGAGAAAGGAGAGAGAACGCAAGGA GAGGC ACGCGAGG GAGG AAA GGC AGGATACCGTTCGTATAGCATACATTATACGAAGTTATGAATTC
		ErmR-2c	GAGGCTCCTGTCACTGCTTCGCTCTGCTTCGGTGTCGTCGCCGTATCTGTGCTC TCTCTACCGTTCGTATAATGTATGCTATACGAAGTTATCTCGAG
SPβ *yok*I::*kan*	Forward Flanking	yokI-5pL	ATCCTCCATTGCTTTAGTCAGTATG
		yokI-1_R	GATTATATCACATTATCCATTAAAAATCAAACCATTTCATTCTCCTTTCAAGCC
	Reverse Flanking	yokI-4_F	GGATGAATTGTTTTAGTACCTAGAAACTTTAGAAAGTAGGTGCG
		yokI-3pR	ACTGAAGACAAACTCCTCAAACG
phi3T *yok*I::*kan*	Forward Flanking	phi3T-1m	GCAATGTTTCCTGAACAGATTTGC
		phi3T-2c	GATTATATCACATTATCCATTAAAAATCAAAATCATTCTCCTTCCATTCTTACTC
	Reverse Flanking	phi3T-3m	GGATGAATTGTTTTAGTACCTAGACACAGGCCGAAGCTGAAGATTGG
		phi3T-4c	CTTGCCTACAACCTCCGCTTC
SPβ *aim*R::erm		AimR-SPB-24mB	CGCGGATCCTATACAATGGCGCTGAGATCC
		AimR-SPB-14cS	ACGCGTCGACCACAAAATGTATTAGGGATCTAAAATGCGG
SPβ *aim*P::erm		AimP-SPB-1mB	CGCGGATCCGACAAAGGCAGCAAGAAGTGC
		AimP-SPB-4c	ATTGTGATGCCACGTTTGACC
SPβ *yop*N::erm		Spbeta_5_S_F	CTGCAGGTCGACACCTGAAATGAATTCTTTCTCAAG
		YopMNO_R	GCCTTTCACCTCATGTCATGTTGC
SPβ *yop*R::erm		YopR_F	CTTCACAGAAACGGATATGAGAG
		YopR_R	CTCTCCCTTGAAACAAAGTAGG
phi3T *aim*R::*erm*	Forward Flanking	AimR-phi3T-1m	CGAATCGTGGAGAAACTTTGCAAATG
		AimR-phi3T-2c	GTTCTCTCTCCTTTCTCGCCTGCCTGCTTTAATTTCAATTGTCTCC
	Reverse Flanking	AimR-phi3T-3m	GCGAAGCAGTGACAGGAGCCTCGGTTTGACAAATTTGAAAGGAGGTG
		AimR-phi3T-4c	CAAGACAATCATATGCTTTTTCCAG
phi3T *aim*P::*erm*	Forward Flanking	AimR-phi3T-5m	GTTGCATTTGGCCAATTATGC
		AimR-phi3T-11c	GCCAATAGTTAAGTAGCTGAAAACCTGAAACGCCAGGATATTTGTACTTTCCAA TGCC
	Reverse Flanking	AimR-phi3T-11m	GGCTTTGTGCTTCTTAAATAATGTATGGCGCGCCGCCGCCAAGTGGATTAATTT TGAATCTGATTCAATTATGG
		AimR-phi3T-4c	CAAGACAATCATATGCTTTTTCCAG
**Plasmids**		**Oligonucleotides**	**Sequence (5'-3')**
pJP2340		AimR-SPBeta-1mH	CCCAAGCTTGACTCGTAATGTGATCTATAG
		AimR-SPBeta-2cS	ACGCGTCGACCATTGTCTCACCTCCTTTAAAGTAAAAG
pJP2750		AimR-phi3T-9mS	ACGCGTCGACCTTGAAATTCTGACAACTATGAGG
		AimR-phi3T-10cSphI	ACATGCATGCCCTCCTTTCAAATTTGTCAAACC
pJP2800		YopR_2F	ACGCGTCGACAGGTGTAGTAGACAAGAATGG
		YopR_2R	ACATGCATGCCCATTTAACCAAAATAGTCAAATGGATTTC
**Southern Blot**		**Oligonucleotides**	**Sequence (5'-3')**
SPβ probe		SPBeta-1m	GATAGGCTTACCGAGGTCTTC
		SPBeta-2c	CTAATGGACGGCTGGAGAGGC

Table S5. Primers used in this study, Related to STAR Methods.

Supplemental References:

S1. Burkholder, P. R., and N. H. Giles. (1947). Induced biochemical mutations in *Bacillus subtilis*. Amer. J. Bot. 34:345.

S2. Westers H, Dorenbos R, van Dijl JM, Kabel J, Flanagan T, Devine KM, *et al*. (2003). Genome engineering reveals large dispensable regions in *Bacillus subtilis*. Mol Biol Evol. *20,* 2076–90.

S3. Dean, D. H., J. C. Orrego, K. W. Hutchinson, and H. O. Halvorson. (1976). New temperate bacteriophage for *Bacillus subtilis*, r11. J. Virol. *20,* 509-519.

S4. Koo BM, Kritikos G, Farelli JD, Todor H, Tong K, Kimsey H, Wapinski I, Galardini M, Cabal A, Peters JM *et al*. (2017). Construction and Analysis of Two Genome-Scale Deletion Libraries for *Bacillus subtilis*. Cell Syst. *4,* 291-305.

S5. Carniol K, Ben-Yehuda S, King N, Losick R. (2005). Genetic dissection of the sporulation protein SpoIIE and its role in asymmetric division in *Bacillus subtilis*. J Bacteriol. *187*, 3511–20.
