# Supplementary material for: The arbitrium system controls prophage induction
Source: Curr Biol. 2021 Nov 22;31(22):5037–5045.e3. doi: 10.1016/j.cub.2021.08.072 (PMC8612738; doi:10.1016/j.cub.2021.08.072)
Supplement: Document S2. Article plus supplemental information [file mmc2.pdf]

Article

Current Biology

The arbitrium system controls prophage induction

Graphical abstract

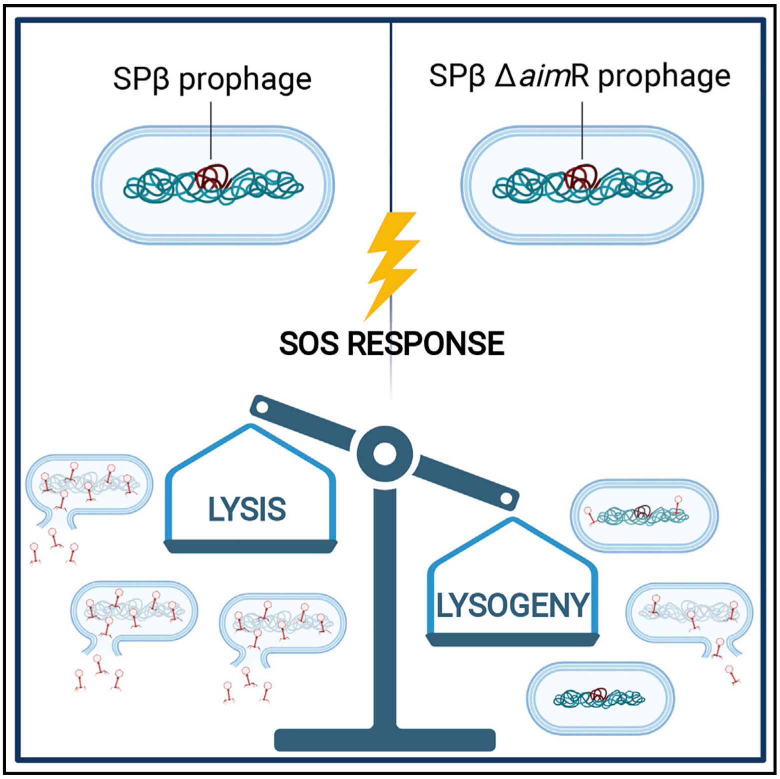

Highlights

- The arbitrium system controls prophage induction in *B. subtilis*
- An operon downstream of the arbitrium system is involved in controlling lysogeny
- The operon is functionally conserved in SPβ-like phages encoding arbitrium systems
- YopR acts as the phage repressor in SPβ

Authors

Aisling Brady, Nuria Quiles-Puchalt, Francisca Gallego del Sol, ..., Wilfried J.J. Meijer, Alberto Marina, José R. Penadés

Correspondence

amarina@ibv.csic.es (A.M.),
j.penades@imperial.ac.uk (J.R.P.)

In brief

Bacillus subtilis phages from the SPβ family use the arbitrium system to communicate during infection of the host. Brady et al. show that this system is also required for induction of the resident prophage after activation of the host SOS response and identify a key operon involved in the control of the lytic/lysogenic cycle.

Brady et al., 2021, Current Biology *31*, 5037–5045
November 22, 2021 © 2021 The Authors. Published by Elsevier Inc.
https://doi.org/10.1016/j.cub.2021.08.072

Current Biology

CellPress
OPEN ACCESS

Article

The arbitrium system controls prophage induction

Aisling Brady,[1,5,6] Nuria Quiles-Puchalt,[1,5,7] Francisca Gallego del Sol,[2] Sara Zamora-Caballero,[2] Alonso Felipe-Ruíz,[2] Jorge Val-Calvo,[3] Wilfried J.J. Meijer,[3] Alberto Marina,[2,8,*] and José R. Penadés[1,4,9,10,*]
[1]Institute of Infection, Immunity and Inflammation, University of Glasgow, Glasgow G12 8TA, UK
[2]Instituto de Biomedicina de Valencia (IBV-CSIC) and CIBER de Enfermedades Raras (CIBERER), 46010 Valencia, Spain
[3]Centro de Biología Molecular “Severo Ochoa” (CSIC-UAM), Universidad Autónoma, Madrid, Spain
[4]MRC Centre for Molecular Bacteriology and Infection, Imperial College London, London SW7 2AZ, UK
[5]These authors contributed equally
[6]Twitter: @AislingBrady95
[7]Twitter: @quiles_nuria
[8]Twitter: @AlbertoMarina14
[9]Twitter: @jrpenades
[10]Lead contact
*Correspondence: amarina@ibv.csic.es (A.M.), j.penades@imperial.ac.uk (J.R.P.)
https://doi.org/10.1016/j.cub.2021.08.072

SUMMARY

Some *Bacillus*-infecting bacteriophages use a peptide-based communication system, termed arbitrium, to coordinate the lysis-lysogeny decision. In this system, the phage produces AimP peptide during the lytic cycle. Once internalized by the host cell, AimP binds to the transcription factor AimR, reducing *aim*X expression and promoting lysogeny. Although these systems are present in a variety of mobile genetic elements, their role in the phage life cycle has only been characterized in phage phi3T during phage infection. Here, using the *B. subtilis* SPβ prophage, we show that the arbitrium system is also required for normal prophage induction. Deletion of the *aim*P gene increased phage reproduction, although the *aim*R deletion significantly reduced the number of phage particles produced after prophage induction. Moreover, our results indicated that AimR is involved in a complex network of regulation and brought forward two new players in the SPβ lysis-lysogeny decision system, YopN and the phage repressor YopR. Importantly, these proteins are encoded in an operon, the function of which is conserved across all SPβ-like phages encoding the arbitrium system. Finally, we obtained mutant phages in the arbitrium system, which behaved almost identically to the wild-type (WT) phage, indicating that the arbitrium system is not essential in the laboratory but is likely beneficial for phage fitness in nature. In support of this, by possessing a functional arbitrium system, the SPβ phage can optimize production of infective particles while also preserving the number of cells that survive after prophage induction, a strategy that increases phage persistence in nature.

INTRODUCTION

Deciphering the basis of communication is essential for understanding the communities where organisms live and their ecological behaviors. The ability to communicate is not restricted to highly evolved animals; bacteria and unicellular eukaryotes also possess sophisticated mechanisms of communication. However, it has recently been described that viruses also have communication mechanisms that allow them to make collective decisions. Quorum-sensing communication mechanisms in bacteriophages (phages)—such as the arbitrium system—to make lysis-lysogeny decisions represent a breakthrough confirming viruses as sophisticated social agents in the microbial world.[1,2] In addition, other social behaviors, such as cooperation, where different viruses co-infect a host,[3,4] or altruism to defeat the CRISPR-Cas-mediated immune defense of bacteria[5,6] confirm that viruses have different communication skills that may have a crucial role in establishing sophisticated social microbial networks.

The novel arbitrium system was described in the *Bacillus subtillis* SPβ group of phages using phi3T as a model. In this elegant system, phages communicate during the infection cycle using a six-amino-acid (aa) peptide (AimP) as a signal.[2] Depending on the concentration of peptide present, phages undergo either a lytic or lysogenic cycle. The arbitrium system is composed of three genes: *aim*P, which encodes the arbitrium peptide; *aim*R, encoding a transcriptional factor that binds to AimP; and *aim*X, which produces a small non-coding RNA that exerts a negative regulatory effect on lysogeny, inducing lysis by a mechanism that has not been deciphered yet.[2] AimP is produced as a 43-aa pro-peptide that is released from the bacterial cell into the surrounding medium. The pro-peptide is then processed into the mature 6-aa AimP before it is imported into neighboring bacteria via the oligopeptide permease (OPP) transporter channel. Once internalized, the mature AimP binds to the AimR receptor and controls its DNA regulatory activity.[2] AimR is a transcriptional factor and, in its apo peptide-free form, promotes *aim*X expression. During the initial stages of infection, when the

© 2021 The Authors. Published by Elsevier Inc.
This is an open access article under the CC BY license (http://creativecommons.org/licenses/by/4.0/).

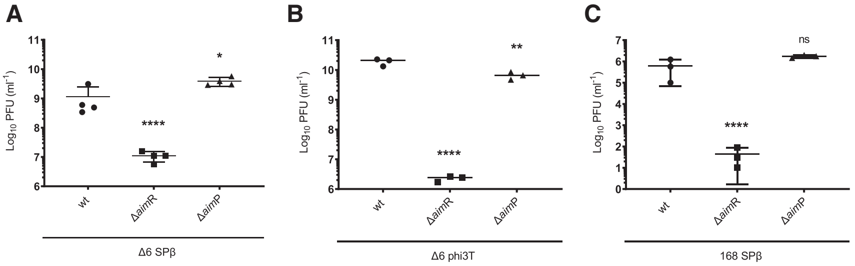

Figure 1. Effect of *aim*R and *aim*P mutations on phage titer

(A) 168 Δ6 strains lysogenic for phage SPβ WT, Δ*aim*R, and Δ*aim*P were MC induced (0.5 μg/mL), and the number of resulting phages were quantified by titering using 168 Δ6 as the recipient strain. The results are represented as the plaque-forming units (PFUs) mL^{-1}. The means and SDs are presented (n = 4). An ordinary one-way ANOVA of transformed data was performed to compare mean differences between SPβ WT, Δ*aim*R, and Δ*aim*P titers. Adjusted p values were as follows: SPβ Δ*aim*R ****$p \leq 0.0001$; SPβ Δ*aim*P *$p = 0.0115$.

(B) 168 Δ6 strains lysogenic for phages phi3T WT, Δ*aim*R, and Δ*aim*P were MC induced (0.5 μg/mL), and the number of resulting phages were quantified by titering using 168 Δ6 as the recipient strain. The results are represented as $PFUs/mL^{-1}$. The means and SDs are presented (n = 3). An ordinary one-way ANOVA of transformed data was performed to compare mean differences between SPβ WT, Δ*aim*R, and Δ*aim*P titers. Adjusted p values were as follows: SPβ Δ*aim*R ****$p \leq 0.0001$; SPβ Δ*aim*P **$p = 0.0058$.

(C) Strain 168 lysogenic for phages SPβ WT, Δ*aim*R, and Δ*aim*P were MC induced (0.5 μg/mL), and the number of resulting phages was quantified by titering using 168 Δ6 as the recipient strain. The results are represented as $PFUs/mL^{-1}$. The means and SDs are presented (n = 3). An ordinary one-way ANOVA of transformed data was performed to compare mean differences between SPβ WT, Δ*aim*R, and Δ*aim*P titers. Adjusted p values were as follows: SPβ Δ*aim*R ****$p \leq 0.0001$; SPβ Δ*aim*P ns, not significant.

number of active phages is low, the arbitrium peptide is absent and AimR activates *aim*X expression, promoting the lytic cycle of the phage (Figure S1). After intense phage replication, AimP will accumulate in the medium, increasing the intracellular concentration of the mature AimP peptide until it reaches the threshold level required to bind to its cognate AimR receptor. When this occurs, AimR cannot activate *aim*X expression. This promotes the lysogenic cycle and the integration of the prophage into the bacterial chromosome, thus preventing eradication of the entire bacterial population by the phage (Figure S1).[2] This simple and elegant communication system allows infecting phages to "decide" between lytic and lysogenic life cycles.

Although the ecological impact of the arbitrium system in phage infection has clearly been established, whether this system has a role in prophage induction or not remains to be determined. Here, we solve this mystery, providing evidence that the arbitrium system has an important role in prophage induction and cellular survival.

RESULTS

Analysis of *aim*R and *aim*P mutants

SPβ is one of the prophages present in the *B. subtillis* 168 strain and was selected as the phage model used to study the impact of the arbitrium system in prophage induction. Several studies have analyzed the transcriptomic landscape of this strain in response to different stimuli, including induction of the SOS response by treating the lysogenic cells with mitomycin C (MC).[7,8] When we analyzed these transcriptomic data in relation to the SPβ prophage, one result raised our curiosity: the expression of the *aim*P gene is relatively high compared to other genes in the uninduced SPβ lysogenic strain,[7,8] suggesting that *aim*P (and therefore the arbitrium system) might have a role during lysogeny. To test this hypothesis, we initially made use of the *B. subtillis* 168 Δ6 strain, in which all the mobile genetic elements (MGEs) present in the original *B. subtillis* 168 strain have been deleted.[9] The strain was lysogenized with SPβ and subsequently used to generate derivative Δ*aim*P or Δ*aim*R mutants. We next tested whether *aim*R or *aim*P impacted SPβ prophage induction. Lysogenic strains carrying either the wild type (WT), the Δ*aim*R, or the Δ*aim*P SPβ prophages were induced with MC, and after leaving them overnight to complete the lysis, the phages present in the lysates were quantified. The titer of the SPβ Δ*aim*P was slightly higher than that observed for the WT SPβ (Figure 1A). Alongside an increased titer, the culture carrying the SPβ Δ*aim*P prophage was significantly more lysed than that carrying the WT prophage after prophage induction (Figure S2A). Importantly, our results also demonstrate that AimR is required for SPβ induction. Thus, after induction, the titer of the SPβ Δ*aim*R phage was significantly reduced compared to the SPβ WT (Figure 1A). Importantly, both the *aim*P and *aim*R mutations could be complemented (Figure S2), confirming the validity of these results.

In support of the role of the arbitrium system during SPβ infection, the plaque morphology of the phages analyzed were different. Although the SPβ *aim*P mutant produced the sharpest plaques, the ones produced by the *aim*R mutants were more diffuse, confirming that the absence of AimP or AimR promotes lysis or lysogeny, respectively (Figure S2D). In fact, when the different lysates were used to analyze lysogenization, the SPβ Δ*aim*R mutant generated more lysogenic cells than the WT after infection of the recipient cells (Figure 4B).

Importantly, and because the *aim*R mutation increases lysogenization, it could be possible that the *aim*R mutant did not generate less infective particles than the WT phage, but these could not be properly quantified because most of the *aim*R mutant phages could integrate after infection. To analyze this possibility, the SPβ WT and Δ*aim*R lysates obtained were used to infect either the *B. subtillis* 168 Δ6 strain or its derivative expressing $aimR_{SP\beta}$. As shown in Figure S3A, although the plaques were sharper in the strain expressing *aim*R (Figure S3C), no differences in the number of plaques formed were observed when the different lysates were plated in either the WT or in the AimR-expressing strain, confirming that *aim*R is required for SPβ prophage induction (Figure 1).

Next, we analyzed whether overexpression of AimR would per se induce the resident SPβ prophage. To do that, we overexpressed the $aimR_{SP\beta}$ gene in the strain lysogenic for SPβ, and after 12 h, we quantified the number of phages present

CellPress
OPEN ACCESS

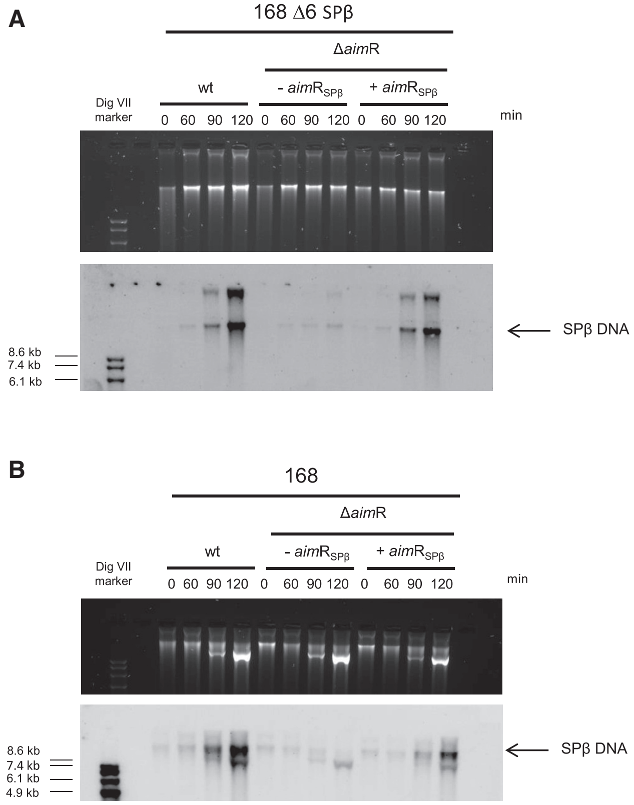

Figure 2. Phage replication of SPβ WT, Δ*aim*R, and Δ*aim*R complemented

(A) Strains Δ6 lysogenic for phages SPβ WT, Δ*aim*R, and Δ*aim*R complemented with *aim*R$_{SPβ}$ were MC induced (0.5 μg/mL), and 1 mL of each culture at different time points after induction was collected. Samples were loaded in a 0.7% agarose gel, Southern blotted, and probed for phage DNA.

(B) Strains 168 lysogenic for phages SPβ WT, ΔaimR, and Δ*aim*R complemented with *aim*R$_{SPβ}$ were MC induced (0.5 μg/mL), and 5 mL of each culture at different time points after induction was collected. Samples were loaded in a 0.7% agarose gel, Southern blotted, and probed for phage SPβ DNA.

in the lysate. We did not observe significant differences between the number of phages obtained from the strain carrying the empty vector versus the one that overexpressed *aim*R (Figure S3D), suggesting that AimR is required once the SOS response has been activated.

Because the arbitrium system played an important role in SPβ, we extended our studies by analyzing the impact of the *aim*R and *aim*P mutations in the phi3T prophage. Note that, although SPβ and phi3T belong to the same family of SPβ phages,[10,11] they encode arbitrium systems that are different in sequence. Our results demonstrated that, in this prophage, the role of AimR seemed to be more relevant, and the titer obtained after induction of the phi3T Δ*aim*R prophage was reduced 10,000 times compared to that seen in the WT phi3T (Figure 1B). Interestingly, and contrary to what is seen with SPβ, the phi3T Δ*aim*P showed a slightly reduced titer after induction, compared to the WT (Figure 1B). Why the *aim*P mutations have different consequences in both phages is an intriguing question that is currently under investigation. Complementation of the *aim*R or *aim*P mutations restored the phage titers, confirming that the observed phenotypes were consequence of the mutations (Figure S2).

Finally, we analyzed the impact of the arbitrium system in prophage induction using a more natural scenario. To do that, we used *B. subtillis* 168 strain, which, in addition to the SPβ prophage, contains 4 other prophages and the integrative conjugative element ICEBs1.[12] We obtained SPβ *aim*R and *aim*P derivative mutants of this strain, and after MC induction of the WT and mutant strains, the SPβ titers were quantified using *B. subtillis* 168 Δ6 as recipient. Note that, in the *B. subtillis* 168 strain, none of the other phages present except SPβ can produce plaques. In support with the fact that ICEBs1 and PBSX (one of the defective prophages present in this strain) interfere with SPβ reproduction,[13,14] the titer of SPβ was significantly reduced (more than 3 logs) after induction of prophage from the *B. subtillis* 168 strain, compared with the induction of the SPβ prophage from *B. subtillis* 168 Δ6 strain (Figure 1). Although the *aim*P mutant did not show any difference in titer, the *aim*R deletion had a more pronounced effect in the *B. subtillis* 168 background compared to what is seen in the *B. subtillis* 168 Δ6 strain, with a reduction in the phage titer higher than 10,000 times (Figure 1C). This result suggests that the arbitrium system may be even more important in strains carrying multiple mobile genetic elements, where these elements compete for resources in terms of induction and transmissibility.

Impact of AimR on phage replication

Although the SPβ master repressor has not yet been identified, the existing results suggested a cascade in prophage activation, starting with the elimination of the SPβ repressor after activation of the cellular SOS response. Once this occurs, the role of AimR in prophage induction turns essential. To acquire a better understanding of what was occurring with the *aim*R mutant, we took samples at different time points of the WT, Δ*aim*R mutant, and complemented SPβ prophages, present in either *B. subtillis* 168 or its derivative Δ6 strain, after MC (SOS) induction of the lysogenic cells and analyzed phage replication. As shown in Figure 2, replication of the SPβ *aim*R mutant was delayed and significantly reduced.

Evolved phage mutants provide insights into AimR function

The previous results suggested that AimR functions either by controlling expression of the genes involved in SPβ replication or by promoting the removal of the phage-encoded master repressor. To gain more of an insight into AimR function, we evolved the SPβ *aim*R mutant in the *B. subtillis* 168 Δ6 background until it produced plaques identical in morphology to those of the WT SPβ phage (see scheme in Figure S4). As previously mentioned, the plaques produced by the SPβ *aim*R mutant have a diffuse (cloudy) morphology (Figure S2D). Different evolved phages, from independent experiments, were obtained and sequenced (Table S1). In three evolved phages, the mutations affected *yop*N, a gene localized in an operon next to the *aim*P gene in the SPβ genome (Figure 3) and encoded a protein with no known function. The mutations identified in the independently evolved phages were different. However, because one of the mutations generated a nucleotide deletion that created an early stop codon in *yop*N, we assumed that, in all cases, the

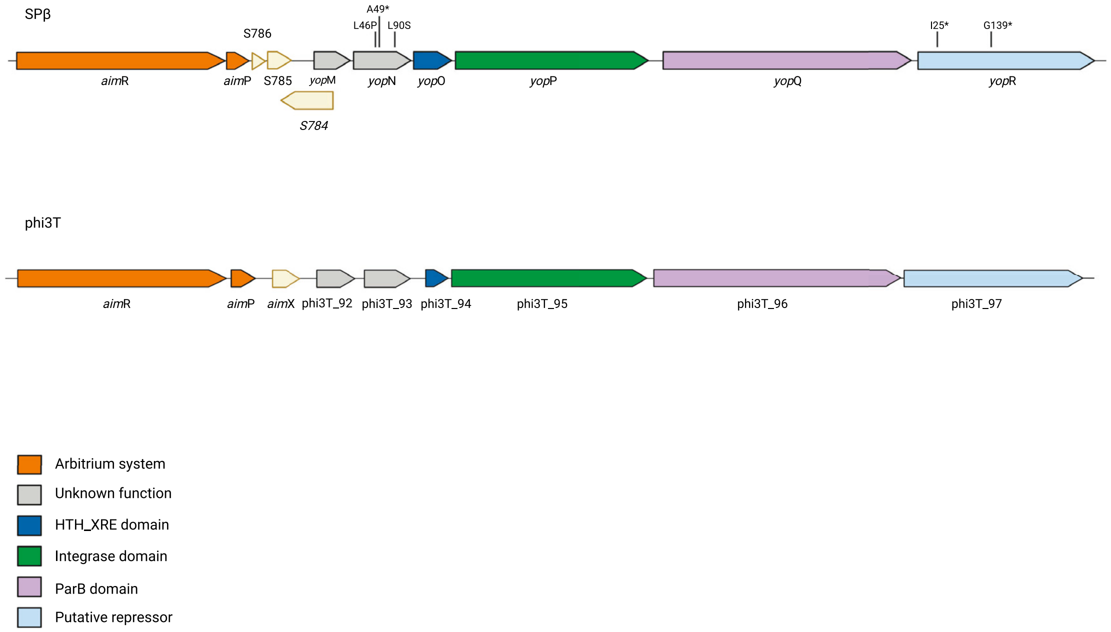

Figure 3. Schematic representation of the SPβ and phi3T arbitrium and operon genetic layout

Diagram shows the genetic organization of the arbitrium genes, *aim*R and *aim*P, followed by the operon directly downstream. Colors denote putative functions according to BLAST results; orange, arbitrium genes; light yellow, sRNAs; gray, unknown function; navy blue, HTH_XRE domain; green, integrase domain; purple, ParB domain; light blue, putative repressor. The mutations obtained during the evolution experiments are marked. Shown was created with BioRender.com. See also Figures S1 and S7 and Tables S1 and S2.

reversion of the *aim*R mutant phenotype was consequence of a loss of function in the YopN protein.

To analyze the behavior of these evolved phages, we lysogenized the *B. subtilis* 168 Δ6 strain with the different evolved phages. Following MC induction of the lysogenic cells, the number of phage particles present in the lysates were quantified. Because the WT and two of the evolved phages have a kanamycin marker inserted in their genome, the number of lysogens obtained after induction of the *B. subtilis* 168 Δ6 derivative strain was also quantified. Note that one of the evolved phages originates from a strain carrying SPβ without a kanamycin marker. In support of the idea that these evolved phages had bypassed the defect generated by the absence of AimR, MC induction of the lysogenic strains carrying these evolved prophages generated phage titers that were significantly higher than that observed for the *aim*R mutant prophage and similar to those observed for the WT SPβ phage (Figure 4A). Interestingly, these evolved phages maintained the ability to lysogenize as observed in the SPβ WT (Figure 4B). Taken together, these results indicated that the evolved phages, which are defective in the arbitrium system, behaved as the WT phage in the lab conditions, suggesting that this system is dispensable in these conditions, but not in nature.

Next, because YopN has no assigned function, and its role in the phage cycle remains undetermined, we generated a *yop*N deletion mutant in the SPβ prophage and tested its impact on the phage cycle. Deletion of *yop*N did not modify the titer of the mutant after MC induction, compared to that of the WT phage (Figure 5). However, the plaques obtained with this mutant were significantly sharper than those obtained with the WT SPβ (Figure S5), with a morphology similar to that generated by the *aim*P mutant (Figure S2D).

Next, and to confirm the results obtained with the evolved phages, we generated a SPβ prophage carrying the double *aim*R/*yop*N deletion and tested its titer after SOS induction of this mutant. As shown in Figure 5, in accordance with the results obtained with the evolved phages, the titer of the double mutant was slightly (but significantly) higher than that observed for the single SPβ Δ*aim*R mutant (Figure 5). The difference observed between the evolved phages and the double SPβ *aim*R/*yop*N are likely to be the consequence of the elimination of the *yop*N coding sequence in the latter, which may have an impact in the stability of the transcript of the operon containing *yop*N. Importantly, and as observed with the single *yop*N mutant, the plaques produced by the double SPβ *aim*R/*yop*N mutant were sharper than those produced either by the WT or the Δ*aim*R SPβ phages (Figure S5). Taken together, these results involve YopN in the process controlling lysis/lysogeny in the SPβ phage.

In addition to the *yop*N mutants, two of the evolved phages characterized in this study presented mutations in *yop*R (Table S1), a gene that is also contained in the same putative operon as *yop*N (Figure 3). The plaques produced by these mutants were even sharper than those produced by the *yop*N mutant, suggesting that these phages had activated their lytic pathway. Confirming this idea, we were not able to obtain lysogens of these evolved phages. Although an initial BLAST analysis showed that YopR has an integrase domain, we propose here that this protein is not required for prophage integration but is the SPβ master repressor. Previous studies have characterized

CellPress
OPEN ACCESS

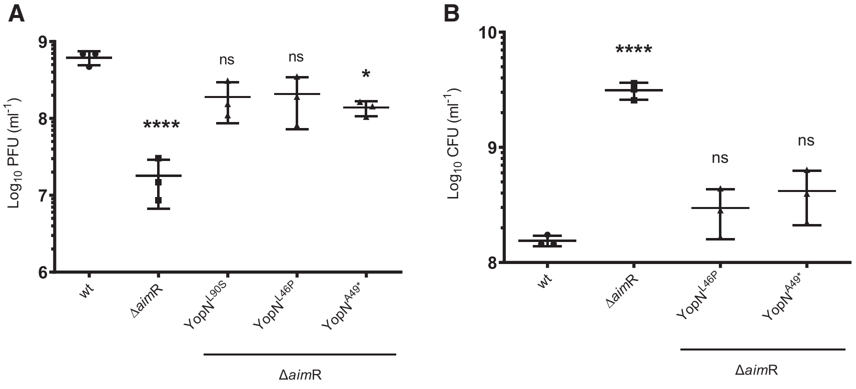

Figure 4. Titer and lysogenization of SPβ WT, Δ*aim*R, Δ*aim*P, and evolved phages

Strains lysogenic for phages SPβ WT, Δ*aim*R, and evolved *aim*R phages were MC induced (0.5 μg/mL).

(A) The number of resulting phages were quantified using 168 Δ6 as the recipient strain. The results are represented as PFUs mL^{-1}. The means and SDs are presented (n = 3). An ordinary one-way ANOVA of transformed data was performed to compare mean differences between titers. Adjusted p values were as follows: SPβ Δ*aim*R ****$p \leq 0.0001$; $YopN^{L90S}$ and $YopN^{L46P}$ ns; $YopN^{A49*}$ *p = 0.0324.

(B) The number of resulting lysogens were quantified using 168 Δ6 as the recipient strain. The results are represented as colony-forming units (CFUs) mL^{-1} normalized by PFUs per milliliter and represented as the log CFU of an average phage titer (1×10^9 PFUs). The means and SDs are presented (n = 3). An ordinary one-way ANOVA of transformed data was performed to compare mean differences in lysogenization. Adjusted p values were as follows: SPβ Δ*aim*R ****$p \leq 0.0001$; $YopN^{L46P}$ and $YopN^{A49*}$ ns.

See also Figure S3 and Table S1.

the SPβ protein SprA, which is the recombinase involved in the integration and excision of the SPβ prophage.[15]

To test the function of YopR, we initially tried to make a *yop*R mutant by inserting an erythromycin marker. The fact that we got a few erythromycin-resistant colonies was unexpected for us, as deletion of the putative phage master repressor would kill the lysogenic cells as a consequence of the induction of the resident prophage. Even more surprising was the fact that the putative *yop*R mutant did not show a reduction in the titer after induction with MC, but it showed plaques with two very distinctive phenotypes, suggesting a mixed population of phages (Figure S5). Concurrently, we tested the original *yop*R::*erm* mutant from the BKE genome-scale deletion library (BKE20790),[16] and we obtained the same mixed population producing two different plaque phenotypes. Although some plaques looked as the WT SPβ prophage, others had the same phenotype previously observed for the evolved SPβ *yop*R mutants (sharper). Because these results suggested the presence of two different phages, we hypothesized that, to maintain integrated the SPβ *yop*R::*erm* prophage, another copy of SPβ would have integrated elsewhere in the bacterial chromosome, complementing the *yop*R mutation. In support of this, PCR analyses confirmed that all the plaques with the cloudy-diffuse morphology carried the WT SPβ phage, although the plaques with the sharp morphology were produced by the SPβ *yop*R mutant. This result suggests that YopR is acting as the repressor of the system, and its deletion abolishes the capacity of the phage to remain integrated as a prophage.

Alternatively, we tried to generate a *yop*R mutant by introducing a second copy of *yop*R elsewhere in the chromosome of the lysogenic strain. Using this strain, we were able to delete *yop*R from the SPβ genome. This mutant phage remained inactive and integrated in the lysogenic strain, because of the complementation with the ectopic copy of *yop*R. However, when this strain was MC induced, the analysis of the lysate showed that these phages produced sharp and clear plaques, as observed with the double *aim*R-*yop*R mutant, and it was unable to lysogenize. Finally, to clearly confirm the role of YopR as the SPβ repressor, we tried to infect the *B. subtilis* 168 Δ6 strain expressing *yop*R with the SPβ phage. As expected for the function of a master repressor, YopR expression in the recipient cells completely blocked plaque formation but increased the number of lysogens generated after infection of the WT or the *yop*R mutant (Figure 6). In summary, these results indicate that AimR is required to remove YopR repression. The molecular details of this interaction and control are currently under study.

The structure of the *yop*N-*yop*R operon is conserved in phages that use the arbitrium system

Because *yop*N and *yop*R seem to belong to the same operon, we scrutinized the rest of the genes forming this transcriptional unit. Four additional genes were annotated in the SPβ genome: *yop*M; *yop*O; *yop*P; and *yop*Q (Figure 3). As occurred with YopN, YopM is a hypothetical protein with no assigned function, YopO seems to be a transcriptional regulator of the Xre family, YopP has a XerC superfamily integrase domain, and YopQ has a ParB_N_Srx superfamily domain (Table S2). Importantly, this operon was uniquely found in *B. subtilis* phages belonging to the SPβ-like family that encode the arbitrium system.

When we examined the genetic layout of the SPβ-like phages carrying the arbitrium system,[2,10] we discovered that, in the majority of the cases, phages carrying this operon also encoded an AimR almost identical to that expressed by SPβ (Table S2). In the exceptions where this did not occur (Table S2), these phages encoded a chimeric AimR, carrying an N-terminal region, responsible of the recognition of the AimR boxes in the phage genome,[17] identical to that present in the SPβ AimR. However, the rest of the protein, involved in AimP recognition, was different, suggesting that these phages encode a different AimP (Figure S6). In support of these ideas, we were able to identify the SPβ AimR boxes in all the analyzed phage genomes carrying the *yop*N-*yop*R operon, although the putative AimP peptide produced by those phages expressing the chimeric AimR was different to that produced by SPβ (GMPRGA versus GIVRGA; mature peptide sequence).

We next scrutinized the region localized 3′ of *aim*P in the phi3T genome to see what genes were located there. As with SPβ, phi3T also carries in this region an operon composed of 6 genes (phi3T_92 to phi3T_97; Figure 3). Importantly, although different in sequence, the proteins encoded by this operon have identical

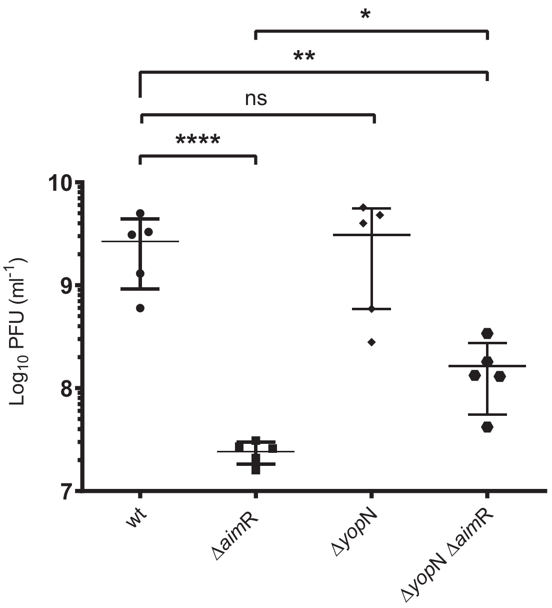

Figure 5. Titer of SPβ WT, Δ*aim*R, Δ*yop*N, and double mutant Δ*aim*R-*yop*N

Strains lysogenic for phages SPβ WT, Δ*aim*R, Δ*yop*N, and Δ*aim*R-*yop*N were MC induced (0.5 μg/mL). The number of resulting phages were quantified using 168 Δ6 as the recipient strain. The results are represented as PFUs mL^{-1}. The means and SDs are presented (n = 5). An ordinary one-way ANOVA of transformed data was performed to compare mean differences between titers. Adjusted p values were as follows: SPβ WT versus SPβ Δ*aim*R ****$p \leq 0.0001$; SPβ Δ*yop*N ns; SPβ Δ*yop*N Δ*aim*R **p = 0.0010. SPβ Δ*aimR* versus SPβ Δ*yop*N Δ*aim*R *p = 0.0351. See also Figures S2, S3, and S5.

predicted functions than those encoded by SPβ (Table S2; Figure 3). In light of these results, we decided to analyze in more detail whether this region was conserved in phages carrying different arbitrium systems. In our analysis, we were able to identify at least 9 families of SPβ-like phages carrying different arbitrium systems with differentiated AimR and AimP genes. After analyzing representatives of each family where the complete sequence of the phage is available, we determined that the genes downstream form part of an operon with genes with conserved functions in the same position as seen for SPβ and phi3T (Table S2; Figure S7). These analyses suggest that this genetic organization and gene composition are important for arbitrium function and prophage induction.

The arbitrium system increases survival of the lysogenic cells after induction

The fact that the SPβ *aim*P mutant, compared to the WT phage, showed higher levels of phage titer after induction raised an interesting question: why is *aim*P highly expressed in the SPβ lysogen? Because *aim*P expression reduces prophage induction, one would expect that this gene would not be expressed during lysogeny. Because AimP expression during infection protects cells from phage killing by promoting lysogenization,[2] we hypothesized that *aim*P expression could also increase cell survival after prophage induction by limiting prophage activation. To test this, we measured cell growth after MC induction of the lysogenic cells carrying either the WT, the Δ*aim*R, or the Δ*aim*P SPβ prophages. As shown in Figure 7, the growth of the different lysogenic strains was inversely proportional to the ability of the different prophage to be SOS induced: thus, the number of lysogenic cells carrying the Δ*aim*R prophage were almost not affected by the induction of the mutant prophage, although the induction of the Δ*aim*P prophage significantly reduced the number of the lysogenic cells (Figure 7). In summary, the arbitrium system provides an interesting equilibrium between prophage induction and cellular survival by providing an almost optimal prophage induction by preserving better the population of lysogenic cells. In other words, our results indicate that the arbitrium system provides a "bet-hedging" strategy that retains some active lysogens during stress conditions.

DISCUSSION

The results presented here demonstrate that the arbitrium system is not only required for phage infection but is also essential for prophage induction. Interestingly, we were able to obtain laboratory-evolved phages that bypassed the necessity to encode an arbitrium system. These results imply that the arbitrium system is not the primary mechanism controlling lysogeny and lysis. However, and mirroring the results obtained during infection, our results demonstrate that the arbitrium system provides an essential ecological role *in vivo* after prophage induction by providing a mechanism that promotes efficient phage reproduction linked to an increased survival of the bacterial population. Because lysogenic cells also contain the intact prophage, by protecting the cells from the lysis that would occur after the activation of the lytic cycle, the arbitrium system provides phages with two alternative and complementary strategies to persist in nature, either as infective particle or as a prophage. One can hypothesize that, when the cellular damage is intense, provoking cell death, an increased production of infective particles could be a better strategy for the phage. However, it is likely that, in many scenarios and after induction of the SOS response, the non-lysogenic cells would be able to repair the damage. However, in this scenario, the presence of a very active and induced prophage would be detrimental for the population by promoting their lysis in circumstances where the cellular damage would be able to prevent the death of the cells. An example of this scenario occurs in the interaction between *Streptococcus pneumoniae* and *Staphylococcus aureus*, where the hydrogen peroxide produced by *S. pneumoniae* kills lysogenic *S. aureus* cells, after activation of the resident prophage, but not the non-lysogenic ones.[18] In this scenario, the presence of an arbitrium system would minimize the damage created by the activation of the resident prophage.

We have yet to decipher how the arbitrium system works mechanistically, but we have been able to identify two additional players in this intriguing system. One is YopN. Although this protein does not seem to have a role in the induction of the WT prophage (at least in the laboratory conditions), this mutant behaves as the *aim*P mutant during infection. The evidence that YopN is an important player of the arbitrium system came from the results from the evolved phages, which indicated that the *yop*N mutation compensates the Δ*aim*R defect. The second player identified corresponds to YopR, which works as the master repressor of the phage. In this moment, we cannot anticipate how AimR, YopN, and YopR interact. Because *yop*N and *yop*R are part of

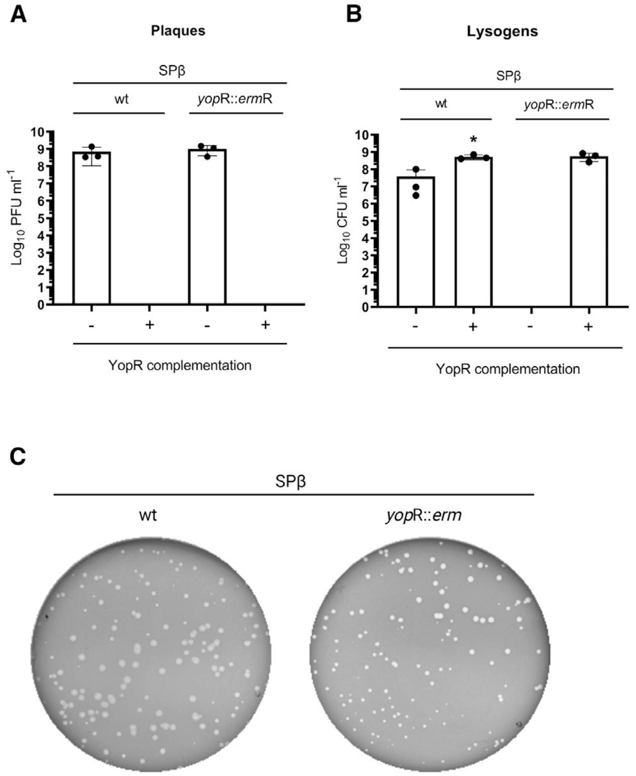

Figure 6. Titer and lysogenization of SPβ WT and *yop*R mutant

Strains lysogenic for phages SPβ WT and SPβ *amy*E::Pspank-YopR *yop*R::*erm*R were MC induced (0.5 μg/mL).

(A) The number of resulting phages were quantified using 168 Δ6 or 168 Δ6 *amy*E::Pspank-YopR as the recipient strain. The results are represented as PFUs mL^{-1}. The means and SDs are presented (n = 3).

(B) The number of resulting lysogens were quantified using 168 Δ6 or 168 Δ6 *amy*E::Pspank-YopR as the recipient strain. The results are represented as CFUs mL^{-1}. The means and SDs are presented (n = 3). An ordinary one-way ANOVA of transformed data was performed to compare mean differences between SPβ lysogen titers obtained using 168 Δ6 or 168 Δ6 *amy*E::Pspank-YopR recipient strains (adjusted p = 0.0171).

(C) The lysates were titered using 168 Δ6 as the recipient strain. The resulting plaque morphologies were photographed. Shown was created with BioRender.com.

See also Figure S5.

an operon containing additional genes (Figure 3), our hypothesis is that some of these genes would be also involved in prophage induction. Therefore, our current working hypothesis is AimR controls the expression of additional gene/s encoding protein/s that, by modulating YopN function, remove YopR activity and induce the lytic cycle of the phage after prophage induction (Figure S1B). In support of this idea, we have previously shown that, in addition to *aim*X promoter, AimR plasticity allows the recognition of additional phage operators. Remarkably, one of the operators proved to be specifically recognized by AimR maps between *yop*S and *yop*R genes,[17] suggesting a direct role in the control of the expression of these genes.

Another interesting feature of the phages carrying the arbitrium system is that they required the activation of the SOS response for induction. Classically, phages have sensed the cellular SOS response by encoding repressors that mimic LexA structure. These repressors, exemplified by the λ cI or the P22 c2, are recognized by the activated RecA* protein that appears as a consequence of the cellular damage, activating the autocleavage and the eliminations of these repressors.[19] Surprisingly, the analysis of SPβ and phi3T genomes failed to reveal open reading frames (ORFs) with the cI architecture, which has precluded the assignment of a putative repressor for these phages. Moreover, it seems a general feature for SPβ-like phages because we have not been able to detect cI-like repressors in other members of this family, indicating that these phages must encode for a different type of repressor. Interestingly, the results of the present work points to YopR as the putative repressor for the SPβ phage family. YopR sequence analysis by PFAM or SMART servers does not find any match with annotated domains, even with low confidence or those annotated as unknown function (DUF). Therefore, it seems that YopR lacks the characteristic motifs and fold of cI repressor. However, our *in silico* analyses with different structural prediction software (Phyre2, Robetta, and RaptorX) confirm this fact, proposing for this protein an architecture with structural homology to the tyrosine recombinase superfamily that includes different families of integrases, transposases, and recombinases.[20] The confidence scores of these models are higher at the N-terminal portion, which corresponds to the integrase core-binding domain, than the C-terminal portion corresponding to the catalytic domain. The low confidence of the C-terminal domain hampers to locate the putative catalytic elements, including the conserved Tyr residue that covalently binds to DNA and is usually placed at the most C-terminal portion of the enzyme. Therefore, from the models, it is not possible to discern whether YopR could act as a functional integrase. In any case, the function of YopR as an active integrase seems not to be required because SPβ encodes SprA, whose genetic and enzymatic characterization has confirmed as the SPβ prophage integrase/excisionase,[15] suggesting an alternative function for YopR. Conversely, the high confidence of the models for N-terminal, core-binding domain supports the DNA-binding capacity of YopR. This domain presents a four-helix bundle fold that includes a prototypical helix-turn-helix DNA-binding motif that mediates the interaction at the integration sites. It has been shown that different integrases have repressor capacity by binding their own promoters.[21,22] For the integrase of P4, this capacity has been restricted to the integrase N-terminal portion,[21] supporting our proposed repressor activity for YopR. Importantly, in this new system, how the SOS response promotes the elimination of this repressor remains to be determined.

Our observations also open an interesting possibility involving the arbitrium system in phage interference. We have demonstrated that AimP expression may have an impact by protecting the lysogenic cells of a massive prophage induction. Another possibility is that this expression may protect the lysogenic cells from an attack of a different phage encoding an AimR protein, which activity would be blocked by the AimP peptide expressed from the lysogen. In this scenario, the presence of AimP would promote lysogenization of the infecting phage, preserving both the lysogenic cells and the resident prophage. Although it is assumed that there is not crosstalk among different arbitrium systems, with the reduced number of studies that have analyzed we cannot discard completely that this process exists in nature.

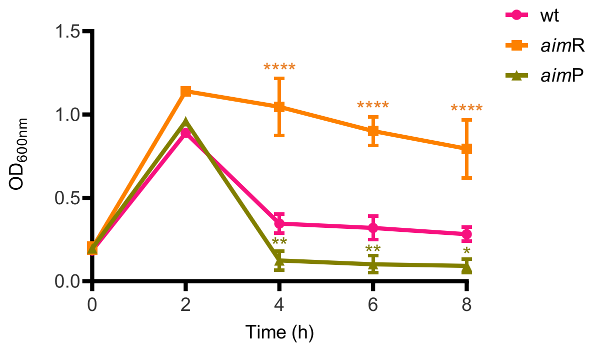

Figure 7. Growth curves of SPβ WT, Δ*aim*R, and Δ*aim*P after MC induction

Strains lysogenic for phages SPβ WT, Δ*aim*R, and Δ*aim*P were MC induced (0.5 μg/mL). Optical density 600 nm (OD_{600nm}) was monitored over time, and cells were collected at time points 0, 2, 4, 6, and 8 h. The means and SDs are presented (n = 3). A two-way ANOVA was performed to compare mean differences in OD_{600nm} values. Adjusted p values were as follows: time 4 h SPβ Δ*aim*R ****p ≤ 0.0001, SPβ Δ*aim*P **p = 0.0077; time 6 h SPβ Δ*aim*R ****p ≤ 0.0001, SPβ Δ*aim*P **p = 0.0085; time 8 h SPβ Δ*aim*R ****p ≤ 0.0001, SPβ Δ*aim*P *p = 0.0226.

The existence of communication systems in phages and other MGEs represents a paradigm shift requiring investigation. Here, we have provided insights into the molecular basis of this novel concept, providing knowledge that we anticipate will be relevant not just for understanding this specific system but also for many biological and evolutionary processes, including the emergence of virulent and multi-resistant bacterial clones. Of note is the fact that not only phages but also plasmids and other MGEs encode arbitrium systems.[10]

STAR★METHODS

Detailed methods are provided in the online version of this paper and include the following:

- KEY RESOURCES TABLE
- RESOURCE AVAILABILITY
  - Lead contact
  - Materials availability
  - Data and code availability
- EXPERIMENTAL MODEL AND SUBJECT DETAILS
  - Strain construction
- METHOD DETAILS
  - Plasmids and cloning
  - Bacteriophage induction assay
  - Bacteriophage titering assay
  - Lysogenisation assays
  - Southern blotting
  - Bioinformatic analyses
- QUANTIFICATION AND STATISTICAL ANALYSIS
  - Statistical analysis

SUPPLEMENTAL INFORMATION

Supplemental information can be found online at https://doi.org/10.1016/j.cub.2021.08.072.

ACKNOWLEDGMENTS

This work was supported by grants MR/M003876/1, MR/V000772/1, and MR/S00940X/1 from the Medical Research Council (UK); BB/N002873/1, BB/V002376/1, and BB/S003835/1 from the Biotechnology and Biological Sciences Research Council (BBSRC, UK); ERC-ADG-2014 proposal no. 670932 Dut-signal (from EU); and Wellcome Trust 201531/Z/16/Z to J.R.P. and grants PID2019-108541GB-I00 from Spanish government (Ministerio de Ciencia e Innovación) and PROMETEO/2020/012 from Valencian government to A.M. A.F.-R. is the recipient of fellowship FPU19/00433 from Ministerio de Ciencia, Innovación y Universidades (Spanish government). Some figures in this manuscript have been created with BioRender.com.

AUTHOR CONTRIBUTIONS

A.M. and J.R.P. conceived the study; A.B., N.Q.-P., F.G.d.S., S.Z.-C., A.F.-R., and J.V.-C. conducted the experiments; and A.B., N.Q.-P., F.G.d.S., S.Z.-C., A.F.-R., J.V.-C., W.J.J.M., A.M., and J.R.P. analyzed the data. A.B., N.Q.-P., and J.R.P. wrote the manuscript.

DECLARATION OF INTERESTS

The authors declare no competing interests.

Received: March 17, 2021
Revised: July 2, 2021
Accepted: August 31, 2021
Published: September 24, 2021

REFERENCES

1. Silpe, J.E., and Bassler, B.L. (2019). A host-produced quorum-sensing autoinducer controls a phage lysis-lysogeny decision. Cell *176*, 268–280.e13.
2. Erez, Z., Steinberger-Levy, I., Shamir, M., Doron, S., Stokar-Avihail, A., Peleg, Y., Melamed, S., Leavitt, A., Savidor, A., Albeck, S., et al. (2017). Communication between viruses guides lysis-lysogeny decisions. Nature *541*, 488–493.
3. Corey, L., Wald, A., Celum, C.L., and Quinn, T.C. (2004). The effects of herpes simplex virus-2 on HIV-1 acquisition and transmission: a review of two overlapping epidemics. J. Acquir. Immune Defic. Syndr. *35*, 435–445.
4. Xue, K.S., Hooper, K.A., Ollodart, A.R., Dingens, A.S., and Bloom, J.D. (2016). Cooperation between distinct viral variants promotes growth of H3N2 influenza in cell culture. eLife *5*, e13974.
5. Borges, A.L., Zhang, J.Y., Rollins, M.F., Osuna, B.A., Wiedenheft, B., and Bondy-Denomy, J. (2018). Bacteriophage cooperation suppresses CRISPR-Cas3 and Cas9 immunity. Cell *174*, 917–925.e10.
6. Landsberger, M., Gandon, S., Meaden, S., Rollie, C., Chevallereau, A., Chabas, H., Buckling, A., Westra, E.R., and van Houte, S. (2018). Anti-CRISPR phages cooperate to overcome CRISPR-Cas immunity. Cell *174*, 908–916.e12.
7. Nicolas, P., Mäder, U., Dervyn, E., Rochat, T., Leduc, A., Pigeonneau, N., Bidnenko, E., Marchadier, E., Hoebeke, M., Aymerich, S., et al. (2012). Condition-dependent transcriptome reveals high-level regulatory architecture in *Bacillus subtilis*. Science *335*, 1103–1106.
8. Buescher, J.M., Liebermeister, W., Jules, M., Uhr, M., Muntel, J., Botella, E., Hessling, B., Kleijn, R.J., Le Chat, L., Lecointe, F., et al. (2012). Global network reorganization during dynamic adaptations of *Bacillus subtilis* metabolism. Science *335*, 1099–1103.
9. Westers, H., Dorenbos, R., van Dijl, J.M., Kabel, J., Flanagan, T., Devine, K.M., Jude, F., Seror, S.J., Beekman, A.C., Darmon, E., et al. (2003). Genome engineering reveals large dispensable regions in *Bacillus subtilis*. Mol. Biol. Evol. *20*, 2076–2090.
10. Stokar-Avihail, A., Tal, N., Erez, Z., Lopatina, A., and Sorek, R. (2019). Widespread utilization of peptide communication in phages infecting soil and pathogenic bacteria. Cell Host Microbe *25*, 746–755.e5.

CellPress
OPEN ACCESS

11. Dragoš, A., Priyadarshini, B., Hasan, Z., Strube, M.L., Kempen, P.J., Maróti, G., Kaspar, C., Bose, B., Burton, B.M., Bischofs, I.B., and Kovács, Á.T. (2021). Pervasive prophage recombination occurs during evolution of spore-forming Bacilli. ISME J. *15*, 1344–1358.

12. Kohm, K., and Hertel, R. (2021). The life cycle of SPβ and related phages. Arch. Virol. *166*, 2119–2130.

13. Johnson, C.M., Harden, M.M., and Grossman, A.D. (2020). An integrative and conjugative element encodes an abortive infection system to protect host cells from predation by a bacteriophage. Biorxiv, 2020.12.13.422588.

14. Martin, M., Dragoš, A., Hölscher, T., Maróti, G., Bálint, B., Westermann, M., and Kovács, Á.T. (2017). De novo evolved interference competition promotes the spread of biofilm defectors. Nat. Commun. *8*, 15127.

15. Abe, K., Takamatsu, T., and Sato, T. (2017). Mechanism of bacterial gene rearrangement: SprA-catalyzed precise DNA recombination and its directionality control by SprB ensure the gene rearrangement and stable expression of spsM during sporulation in *Bacillus subtilis*. Nucleic Acids Res. *45*, 6669–6683.

16. Koo, B.-M., Kritikos, G., Farelli, J.D., Todor, H., Tong, K., Kimsey, H., Wapinski, I., Galardini, M., Cabal, A., Peters, J.M., et al. (2017). Construction and analysis of two genome-scale deletion libraries for *Bacillus subtilis*. Cell Syst. *4*, 291–305.e7.

17. Gallego Del Sol, F., Penadés, J.R., and Marina, A. (2019). Deciphering the molecular mechanism underpinning phage arbitrium communication systems. Mol. Cell *74*, 59–72.e3.

18. Selva, L., Viana, D., Regev-Yochay, G., Trzcinski, K., Corpa, J.M., Lasa, I., Novick, R.P., and Penadés, J.R. (2009). Killing niche competitors by remote-control bacteriophage induction. Proc. Natl. Acad. Sci. USA *106*, 1234–1238.

19. Little, J.W. (1984). Autodigestion of lexA and phage lambda repressors. Proc. Natl. Acad. Sci. USA *81*, 1375–1379.

20. Jayaram, M., Ma, C.-H., Kachroo, A.H., Rowley, P.A., Guga, P., Fan, H.-F., and Voziyanov, Y. (2015). An overview of tyrosine site-specific recombination: from an Flp perspective. Microbiol. Spectr. *3*, 10.1128/microbiolspec.MDNA3-0021-2014.

21. Piazzolla, D., Calì, S., Spoldi, E., Forti, F., Sala, C., Magnoni, F., Dehò, G., and Ghisotti, D. (2006). Expression of phage P4 integrase is regulated negatively by both Int and Vis. J. Gen. Virol. *87*, 2423–2431.

22. Chittò, M., Berger, M., Berger, P., Klotz, L., Dröge, P., and Dobrindt, U. (2020). IHF stabilizes pathogenicity island I of uropathogenic *Escherichia coli* strain 536 by attenuating integrase I promoter activity. Sci. Rep. *10*, 9397.

23. Burkholder, P.R., and Giles, N.H., Jr. (1947). Induced biochemical mutations in *Bacillus subtilis*. Am. J. Bot. *34*, 345–348.

24. Dean, D.H., Orrego, J.C., Hutchison, K.W., and Halvorson, H.O. (1976). New temperate bacteriophage for *Bacillus subtilis*, rho 11. J. Virol. *20*, 509–519.

25. Carniol, K., Ben-Yehuda, S., King, N., and Losick, R. (2005). Genetic dissection of the sporulation protein SpoIIE and its role in asymmetric division in *Bacillus subtilis*. J. Bacteriol. *187*, 3511–3520.

26. Serrano, E., and Carrasco, B. (2019). Measurement of the length of the integrated donor DNA during Bacillus subtilis natural chromosomal transformation. Bio Protoc. *9*, e3338.

27. Simossis, V.A., and Heringa, J. (2005). PRALINE: a multiple sequence alignment toolbox that integrates homology-extended and secondary structure information. Nucleic Acids Res. *33*, W289–W294.

STAR★METHODS

KEY RESOURCES TABLE

REAGENT or RESOURCE	SOURCE	IDENTIFIER
Chemicals, peptides, and recombinant proteins		
Lysogeny broth (LB), Miller	Sigma – Aldrich	Cat. # L3522-1KG
Lysogeny broth (LB), Lennox	Sigma – Aldrich	Cat. # L3022-1KG
Agar	Formedium	Cat. # AGA02
Spectinomycin dihydrochloride pentahydrate	Sigma – Aldrich	Cat. # S4014-5G
Erythromycin	Sigma – Aldrich	Cat. # E6376-25G
Kanamycin Sulfate	Sigma – Aldrich	Cat. # 60615-5G
Ampicillin Sodium Salt	Sigma – Aldrich	Cat. # A9518-25G
Isopropyl-β-D-thio-galactopyranoside (IPTG)	Melford	Cat. # 156000-5.0
Ammonium sulfate	Sigma – Aldrich	Cat. # A4915-500G
K_2HPO_4	Fisher scientific	Cat. # 10509263
KH_2PO_4	Fisher scientific	Cat. # 10573181
Tri-sodium citrate dihydrate	Fisher scientific	Cat. # 10396430
D-(+)-Glucose	Sigma – Aldrich	Cat. # G7021-1KG
Yeast extract	Fisher scientific	Cat. # 11407541
Casein hydrolysate	Sigma – Aldrich	Cat. # 22090-100G
Magnesium sulfate heptahydrate	VWR	Cat. # 25165.26
L-tryptophan	Sigma – Aldrich	Cat. # T8941-25G
L-methionine	Sigma – Aldrich	Cat. # M9625-25G
$CaCl_2$	VWR	Cat. # 190464K
Manganese II chloride dihydrate	Sigma – Aldrich	Cat. # 1059340100
Mitomycin C	Sigma – Aldrich	Cat. # M0503-5X2MG
NaCl	VWR	Cat. # 27810.295
Tris Base	Fisher scientific	Cat. # 10376743
Gen Elute Bacterial genomic DNA Kit	Sigma – Aldrich	Cat. # NA2120-1KT
Nylon membrane	Sigma – Aldrich	Cat. # 11417240001
Digoxigenin-11-dUTP, alkali-stable	Sigma – Aldrich	Cat. # 11093088910
Anti-Digoxigenin-AP	Sigma – Aldrich	Cat. # 11093274910; RRID:AB_2734716
UltraPure Agarose	Thermo Fisher	Cat. # 16500-500
Lysozyme	Sigma – Aldrich	Cat. # 10837059001
Proteinase K	Sigma – Aldrich	Cat. # P2308-500MG
Experimental models: Organisms/strains		
Bacillus subtilis subsp. *subtilis* str. 168 (1A700)	Burkholder and Giles[23]	Bacillus Genetic Stock Centre (http://bgsc.org)
Bacillus subtilis subsp. *Subtilis* str. 168 (1A700) derivatives (listed in Table S3)	N/A	N/A
Bacillus subtilis subsp. *Subtilis* str. 168 IL26	Dean et al.[24]	Bacillus Genetic Stock Centre (http://bgsc.org)
Bacillus subtilis subsp. *Subtilis* str. 168 IL26 derivatives (listed in Table S3)	N/A	N/A
Bacillus subtilis subsp. *Subtilis* str. 168 Δ6 (1A1299)	Westers et al.[9]	Bacillus Genetic Stock Centre (http://bgsc.org)
Bacillus subtilis subsp. *subtilis* str. 168 Δ6 (1A1299) derivatives (listed in Table S3)	N/A	N/A
Oligonucleotides		
See Tables S5 and S6 for list of oligonucleotides used in this study	N/A	N/A

RESOURCE AVAILABILITY

Lead contact

Further information and requests for resources and reagents should be directed to and will be fulfilled by the lead contact, José R Penadés (j.penades@imperial.ac.uk).

Materials availability

All bacterial strains and plasmids generated during this work are freely available from José R. Penadés (j.penades@imperial.ac.uk). The study did not generate new reagents.

Data and code availability

All data reported in this paper will be shared by the lead contact upon request.

EXPERIMENTAL MODEL AND SUBJECT DETAILS

All bacterial strains used in this study belong to *Bacillus subtilis* or *Escherichia coli* species. *B. subtilis* strains were routinely grown at 37°C on LB (Miller) agar plates or in LB (Miller) broth liquid medium shaking at 200 rpm. *E. coli* DH5α was grown at 37°C on LB (Lennox) agar plates or in LB (Lennox) broth shaking at 180 rpm. When required, antibiotics were utilized at the following concentrations: erythromycin (1 μg ml-1), kanamycin (10 μg ml-1), ampicillin (100 μg ml-1) or spectinomycin (100 μg ml-1).

Strain construction

The SPβ phage (accession number NC_001884) has been recently established in our lab as our model to study arbitrium communication. Bacterial strains used in this study are listed in Table S3. *B. subtilis* strains 168, Δ6, 1L26 (phi3T, accession number KY030782) and the BKE Genome-Scale deletion library mutants were obtained from the Bacillus Genetic Stock Centre (BGSC).

To generate the deletion mutants in phage SPβ, the corresponding mutant strain from the BKE collection was used as a template for a PCR using primers amplifying the desired gene plus 1 Kb of flanking region. In the case of phi3T, we generated overlapping PCRs containing the erythromycin marker (including the lox sites) and 1 Kb of flanking region for the desired gene. These PCRs were transformed into the Δ6 SPβ or phi3T strain and selected for erythromycin. Once the insertion of the erythromycin cassette was confirmed by PCR and sequencing, the antibiotic resistance cassette was removed as previously described[16]. Briefly, plasmid pDR244 was transformed into strains harboring the *lox*P-flanked antibiotic resistance cassette with selection for spectinomycin resistance at 30°C to allow for *cre*/*lox*-mediated loop-out of the cassette. Transformant colonies were then streaked onto LB plates and incubated overnight at 42°C for removal of the temperature-sensitive plasmid. Resulting strains were screened for plasmid curing (loss of spectinomycin resistance) and the antibiotic resistance cassette (loss of erythromycin resistance). Strains were streaked to single colonies and confirmation of the clean mutant was performed using PCR. Similarly, we introduced into the SPβ and phi3T genomes a kanamycin cassette replacing the *yok*I gene that was not essential for the phage, by amplifying the marker without including the *lox* sites from one of the BKK Genome-Scale deletion library mutants (BGSC).

METHOD DETAILS

Plasmids and cloning

Plasmids generated in this study are listed in Table S4. The $AimR_{SP\beta}$ and $AimR_{phi3T}$ and the *yop*R genes were cloned into the *amyE* integration vector pDR110 under the control of the IPTG inducible promoter P_{spank}[25]. Cloning was performed after PCR amplification of the appropriate template DNA using primers listed in Table S5. Competent cell preparation and transformation was performed as described[26]. Briefly, *B. subtilis* cells were grown in GM1 minimum medium to early stationary phase to induce natural competence and 1 μg of plasmid was added and incubated at 37°C for 1 h with shaking at 210 rpm. The culture was centrifuged at 6000 g for 1 min, 800 μL of the supernatant removed, and the pellet re-suspended in 400 μL and plated out onto the relevant antibiotic plates. Plates were incubated at 37°C for 24 h.

Bacteriophage induction assay

For induction, an overnight culture was diluted 1:100 in LB media supplemented with 0.1 mM $MnCl_2$ and 5 mM $MgCl_2$ and then grown at 37°C with 210 rpm shaking until reaching absorbance 0.2 at 600 nm. This step was repeated twice to ensure the cells were in exponential growth. After the second growth Mitomycin C (MC) at 0.5 μg ml^{-1} was added to the culture. Where experiments were performed to test the complementation of the mutants, 1 mM of IPTG was added at the same time as MC induction. The induced cultures were incubated at 30 °C with 80 rpm shaking for 4 h and then left overnight at room temperature. Following lysis, samples were filtered using 0.2 μm filters and lysates were stored at 4°C until use.

Bacteriophage titering assay

The number of phage particles contained in the phage lysate of interest were quantified by a titering assay. An overnight culture of the relevant recipient strain (normally *B. subtilis* Δ6 or with the corresponding integration vector) was diluted 1/100 in LB supplemented with 0.1 mM $MnCl_2$ and 5 mM $MgCl_2$ and then grown at 37°C with 210 rpm shacking until reaching absorbance 0.2 at 600 nm. If needed 0.1 mM IPTG was added. Then, 100 μL of recipient bacteria was infected with 100 μL of serial dilutions of phage lysate in phage buffer (PhB; 1 mM NaCl, 0.05 M Tris pH 7.8, 0.1 mM $MnCl_2$, 5 mM $MgCl_2$) at room temperature for 10 min and 3 mL of phage top agar (LB media supplemented with 0.1 mM $MnCl_2$ and 5 mM $MgCl_2$ and 0.7% agar) at 55°C was added to the culture-phage mix and immediately poured over phage base agar plates (LB media supplemented with 0.1 mM $MnCl_2$ and 5 mM $MgCl_2$ and 1.5% agar). Plaques were counted after overnight growth at 37°C temperature and photographed. To obtain evolved SPβ Δ*aim*R phages, plaque lawns from SPβ Δ*aim*R titrations using Δ6 as a recipient strain were collected and added to 4 mL of PhB followed by centrifugation and filtration to acquire new SPβ Δ*aim*R phage lysates. The resulting lysates were used to infect fresh cultures of recipient bacteria and the process was repeated until plaques showing wt morphology were observed as a majority in the phage population (Figure S4). Individual plaques were isolated and evolved phages were subsequently verified as Δ*aim*R mutants by PCR, titered and sent for whole-genome sequencing (MicrobesNG, University of Birmingham).

Lysogenisation assays

The number of lysogens were quantified by growing a recipient strain to $OD_{600nm} = 0.2$. Lysates of interest that contain the kanamycin marker were serially diluted in PhB and 100 μL was added to 1 mL of the recipient bacteria in 12 mL tubes. The mixture was incubated at 37°C for 30 min to allow the phage to infect bacteria. The bacteria-phage mixture was then transferred to 1.5 mL Eppendorf tubes and centrifuged at 6,600 rpm for 1 min. The supernatant was removed, and the bacterial pellet was resuspended in 400 μL of fresh LB broth before plating onto selective antibiotic LB agar plates. Plates were incubated overnight at 37°C. The number of colony forming units (CFU) was calculated.

Southern blotting

Samples were taken at 0, 60, 90 and 120 min after adding MC (M0503, Sigma-Aldrich). For 168 Δ6 lysogenic strains, 1 mL of sample was pelleted and frozen at −20°C until all samples were obtained. For 168 lysogenic strains 5 mL was pelleted. The samples were processed for total bacterial DNA extraction using Gen Elute Bacterial genomic DNA Kit (NA2120, Sigma-Aldrich). Afterward, 5 μL of sample was mixed with 5 μL of 2X loading dye and run on a 0.7% agarose gel at 25V overnight. DNA was transferred to a nylon membrane (0.45 mm hybond-N pore diameter, Amersham Life Science) and exposed using a DIG-labeled probe (Digoxigenin-11-dUTP alkali-labile, Roche) and anti-DIG antibody (Anti-Digoxigenin-AP Fab fragments, Roche) as per the suppliers' protocol, before washing and visualization with Chemdoc imager. The primers used to obtain the labeled probes are shown in Table S5.

Bioinformatic analyses

Alignment of the $AimR_{SP\beta}$ and $AimR_{KATMIRA1933}$ sequences was performed using the PRALINE[27] server (Figure S6).

QUANTIFICATION AND STATISTICAL ANALYSIS

Statistical analysis

Statistical analysis was performed as indicated in the figure legends. Briefly, phage and lysogenisation titers were log_{10}-transformed and analyzed by a One-Way ANOVA followed by an appropriate multiple comparisons test (Dunnett's or Tukey's). For analysis of AimR overexpression data, titers were log_{10}-transformed and analyzed by a Two-Way ANOVA followed by Bonferroni's multiple comparisons test. All analysis was done using GraphPad Prism 9 software. The p values represented in each figure are shown in the figure legends.

Current Biology, Volume *31*

Supplemental Information

The arbitrium system controls prophage induction

Aisling Brady, Nuria Quiles-Puchalt, Francisca Gallego del Sol, Sara Zamora-Caballero, Alonso Felipe-Ruíz, Jorge Val-Calvo, Wilfried J.J. Meijer, Alberto Marina, and José R. Penadés

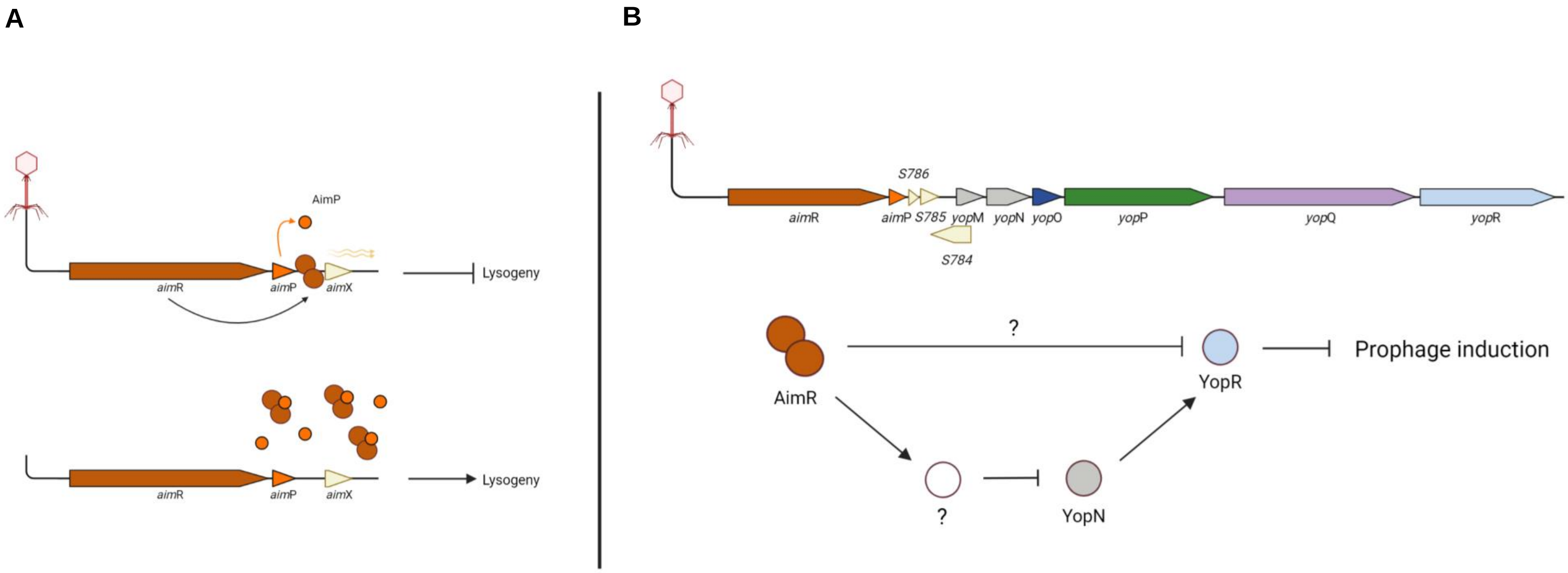

Figure S1. Model for the mechanism of action of the arbitrium system in phages of the SPβ family, Related to Figure 3. (A) The arbitrium model prior to this study describes that after infection of a SPβ prophage, AimR is being expressed and binds to the operator site promoting expression of the *aim*X sRNA and promoting the lytic cycle. After AimP accumulates above the threshold levels, it binds to AimR disrupting its binding to the DNA and reducing expression of *aim*X, leading to lysogeny. (B) Our understanding is that the arbitrium system of SPβ is involved in a more complex mechanism to control prophage induction. The regulator AimR functions to inhibit the phage repressor, YopR, thus promoting prophage induction. Another component of the system is YopN that we hypothesise to promote YopR activity, acting as a negative regulator of prophage induction. We propose that following activation of the SOS response, AimR activates an unknown component that blocks YopN function, thus reducing the activity of YopR and promoting induction.

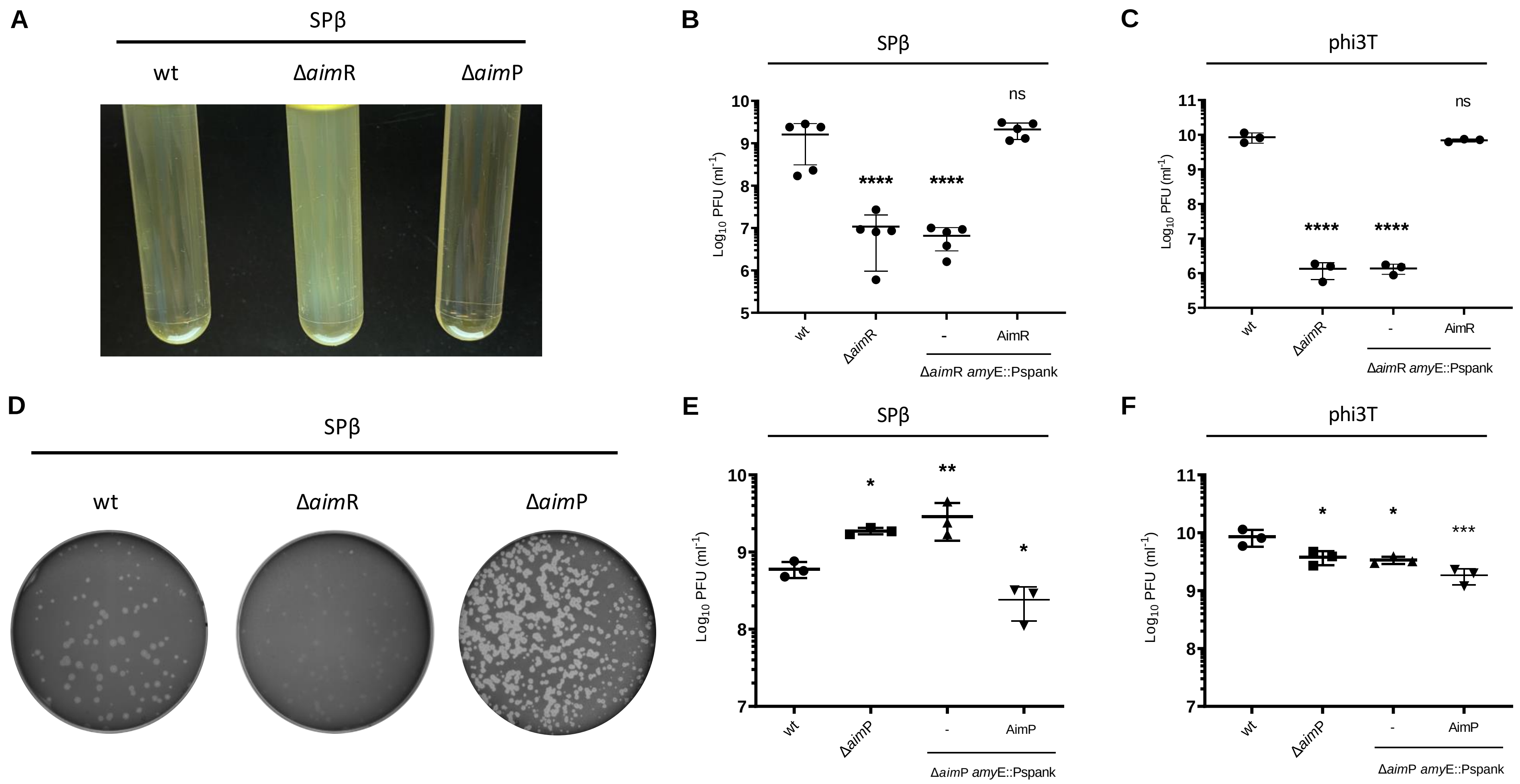

Figure S2. Analysis of the *aim*R and *aim*P deletions in SPβ-type prophages, Related to Figure 5. (A) Lysogenic strains for phage SPβ, SPβ Δ*aim*R or SPβ Δ*aim*P were MC induced (0.5 µg/ml) and incubated at 30 °C with 80 rpm shaking for 4 h. The lysates were left overnight at room temperature before being photographed. (B) Complementation of the *aim*R mutant in SPβ. Strains lysogenic for phage SPβ wt, Δ*aim*R, Δ*aim*R *amy*E::Pspank and Δ*aim*R *amy*E::Pspank-AimR were MC induced (0.5 µg/ml) and the number of resulting phages were quantified by titering using 168 Δ6 as the recipient strain. The results are represented as the plaque forming units (PFUs) mL^{-1}. The means and SDs are presented (n = 5). An ordinary one-way ANOVA of transformed data was performed to compare mean differences between titres. Adjusted p values were as follows: SPβ Δ*aim*R ****p = < 0.0001; Δ*aim*R *amy*E::Pspank ****p = < 0.0001; Δ*aim*R *amy*E::Pspank-AimR ns = not significant. (C) Complementation of the *aim*R mutant in phi3T. Strains lysogenic for phages phi3T wt, Δ*aim*R, Δ*aim*R *amy*E::Pspank and Δ*aim*R *amy*E::Pspank-AimR were MC induced (0.5 µg/ml) and the number of resulting phages were quantified by titering using 168 Δ6 as the recipient strain. The results are represented as PFUs/mL^{-1}. The means and SDs are presented (n = 3). An ordinary one-way ANOVA of transformed data was performed to compare mean differences between titres. Adjusted p values were as follows: SPβ Δ*aim*R ****p = < 0.0001; Δ*aim*R *amy*E::Pspank ****p = < 0.0001; Δ*aim*R *amy*E::Pspank-AimR ns = not significant. (D) SPβ lysates were tittered using 168 Δ6 as the recipient strain. The resulting plaque morphologies were photographed. (E) Strains lysogenic for phage SPβ wt, Δ*aim*P, Δ*aim*P *amy*E::Pspank and Δ*aim*P *amy*E::Pspank-AimP were MC induced (0.5 µg/ml) and the number of resulting phages were quantified by titering using 168 Δ6 as the recipient strain. The results are represented as the plaque forming units (PFUs) mL^{-1}. The means and SDs are presented (n = 3). An ordinary one-way ANOVA of transformed data was performed to compare mean differences between titres. Adjusted p values were as follows: SPβ Δ*aim*P *p = 0.0205; Δ*aim*P *amy*E::Pspank **p = 0.0049; Δ*aim*P *amy*E::Pspank-AimP *p = 0.0391. (F) Strains lysogenic for phages phi3T wt, Δ*aim*P, Δ*aim*P *amy*E::Pspank and Δ*aim*P *amy*E::Pspank-AimP were MC induced (0.5 µg/ml) and the number of resulting phages were quantified by titering using 168 Δ6 as the recipient strain. The results are represented as PFUs/mL^{-1}. The means and SDs are presented (n = 3). An ordinary one-way ANOVA of transformed data was performed to compare mean differences between titres. Adjusted p values were as follows: phi3T Δ*aim*P *p = 0.0220; Δ*aim*P *amy*E::Pspank *p = 0.0125; Δ*aim*P *amy*E::Pspank-AimP ***p = 0.0005.

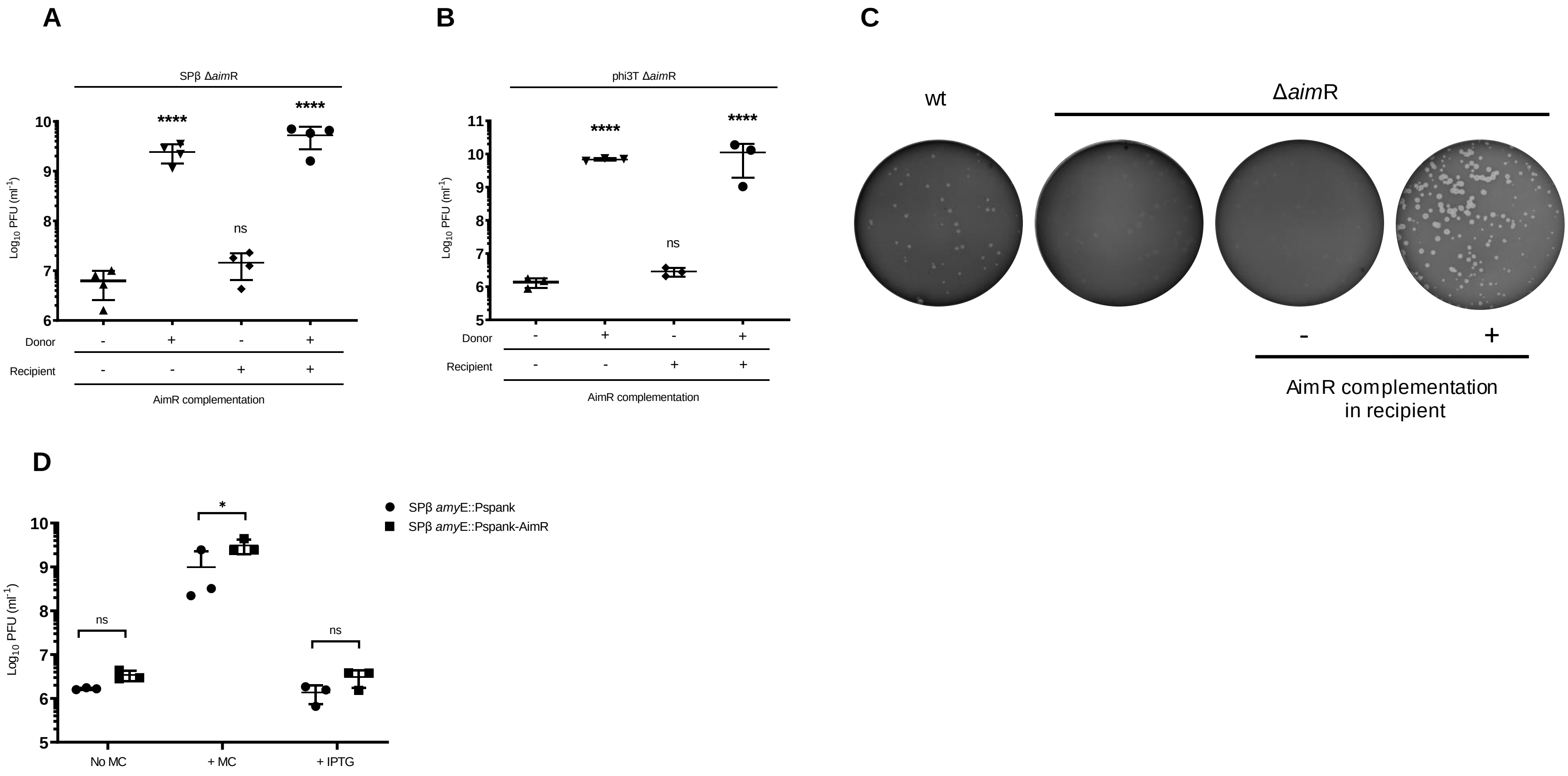

Figure S3. Complementation of the *aim*R mutants in recipient strain , Related to Figure 4 and Figure 5. (A) Strain lysogenic for phage SPβ Δ*aim*R was MC induced (0.5 μg/ml) and the number of resulting phages were quantified by titering using 168 Δ6 *amy*E::Pspank (-) or 168 Δ6 *amy*E::Pspank-AimR$_{SPβ}$ (+) as recipient strains. The results are represented as the plaque forming units (PFUs) mL^{-1}. The means and SDs are presented (n = 4). An ordinary one-way ANOVA of transformed data was performed to compare mean differences between titres. Adjusted p values were as follows: column A vs column B ****p = <0.0001; column A vs column C ns = not significant; column A vs column D ****p = <0.0001. (B) Strain lysogenic for phage phi3T Δ*aim*R was MC induced (0.5 μg/ml) and the number of resulting phages were quantified by titering using 168 Δ6 *amy*E::Pspank (-) or 168 Δ6 and Δ*aim*R *amy*E::Pspank-AimR$_{phi3T}$ (+) as recipient strains. The results are represented as the plaque forming units (PFUs) mL^{-1}. The means and SDs are presented (n = 3). An ordinary one-way ANOVA of transformed data was performed to compare mean differences between titres. . Adjusted p values were as follows: column A vs column B ****p = <0.0001; column A vs column C ns = not significant; column A vs column D ****p = <0.0001. (C) Plaques morphologies produced after titration of the SPβ Δ*aim*R using 168 Δ6 *amy*E::Pspank (-) or 168 Δ6 *amy*E::Pspank-AimR$_{SPβ}$ (+) as recipient strains were photographed. (D) Overexpression of AimR does not induce the lytic cycle. Strains lysogenic for phage SPβ *amy*E::Pspank and SPβ *amy*E::Pspank-AimR$_{SPβ}$ were analysed for their ability to produce phage particles under several conditions: without induction (No MC), with phage induction (+ MC 0.5 μg/ml) and with Pspank induction (+ IPTG 1mM). The number of resulting phages were quantified by titering using 168 Δ6 as the recipient strain. The results are represented as the plaque forming units (PFUs) mL^{-1}. The means and SDs are presented (n = 3). An ordinary one-way ANOVA of transformed data was performed to compare mean differences between titres. Differences in titer with "No MC" and "+ IPTG" were not significant (ns). The adjusted p value comparing SPβ *amy*E::Pspank and *amy*E::Pspank-AimR + MC *p = 0.0227.

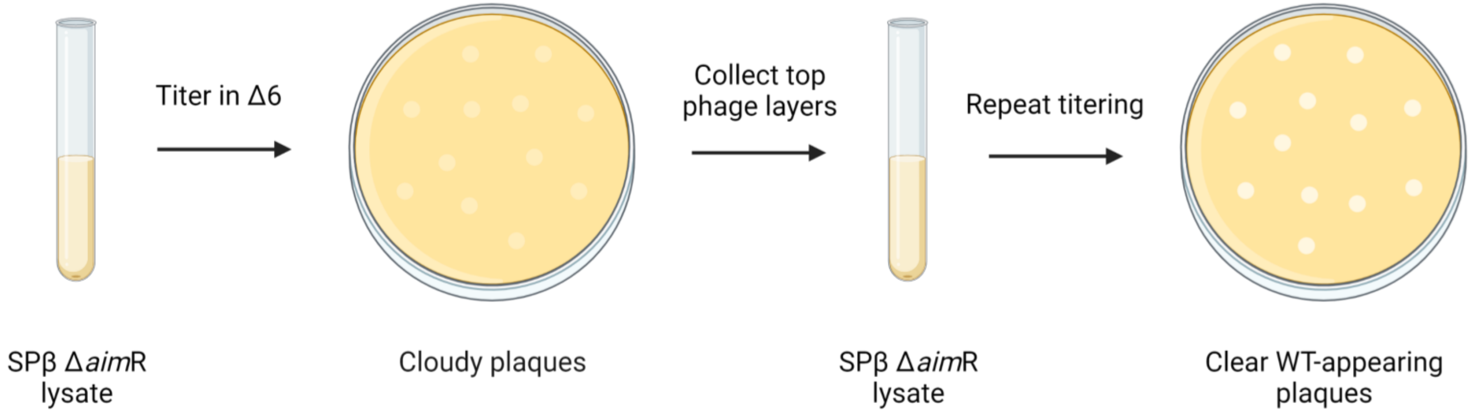

Figure S4. Schematic representation of the SPβ Δ*aim*R evolution procedure Related to STAR Methods. SPβ Δ*aim*R lysate was acquired following MC induction of a lysogenic strain carrying the SPβ Δ*aim*R phage. The lysate was titered using 168 Δ6 as the recipient strain and the resulting cloudy plaques were collected and passaged, as described in the STAR Methods, until wt-appearing plaques were obtained. Created with BioRender.com

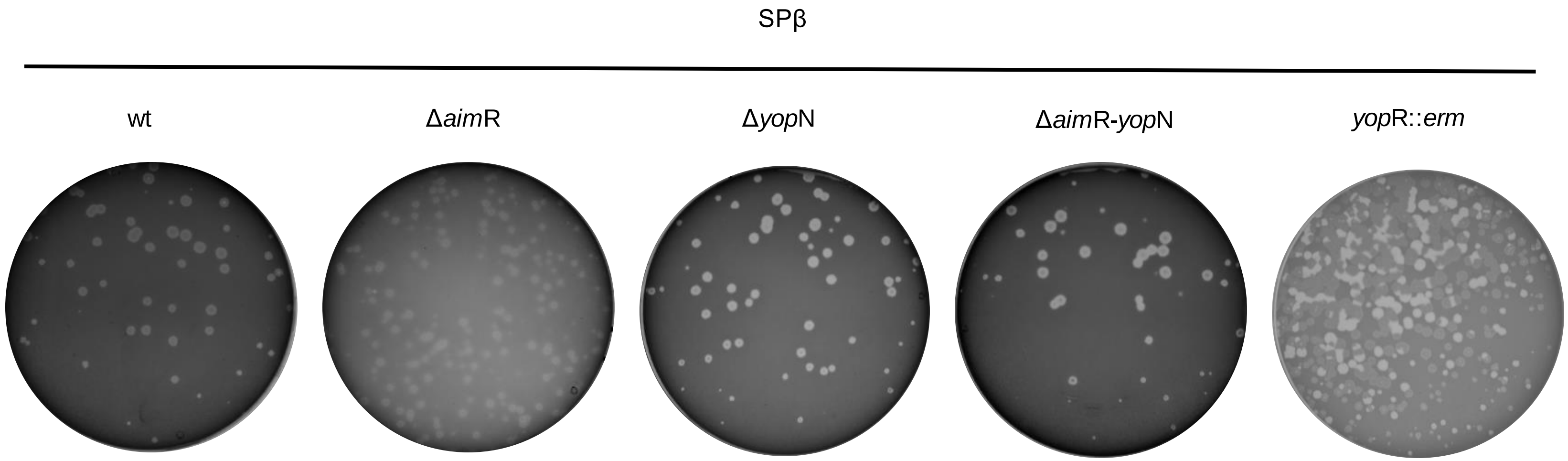

Figure S5. Plaque morphology of SPβ wt, Δ*aim*R, Δ*yop*N, Δ*aim*R-*yop*N and *yop*R::*erm* phages, Related to Figure 5 and Figure 6. Strains lysogenic for phage SPβ wt, Δ*aim*R, Δ*yop*N and Δ*aim*R-*yop*N were MC induced (0.5 μg/ml) and titered using 168 Δ6 as the recipient strain. A strain lysogenic for phage SPβ was transformed with an erythromycin cassette to replace the *yop*R gene. The resulting strain, supposedly *yop*R::*erm*, was MC induced (0.5 μg/ml) and titered using 168 Δ6 as the recipient strain. The resulting plaque morphologies were photographed.

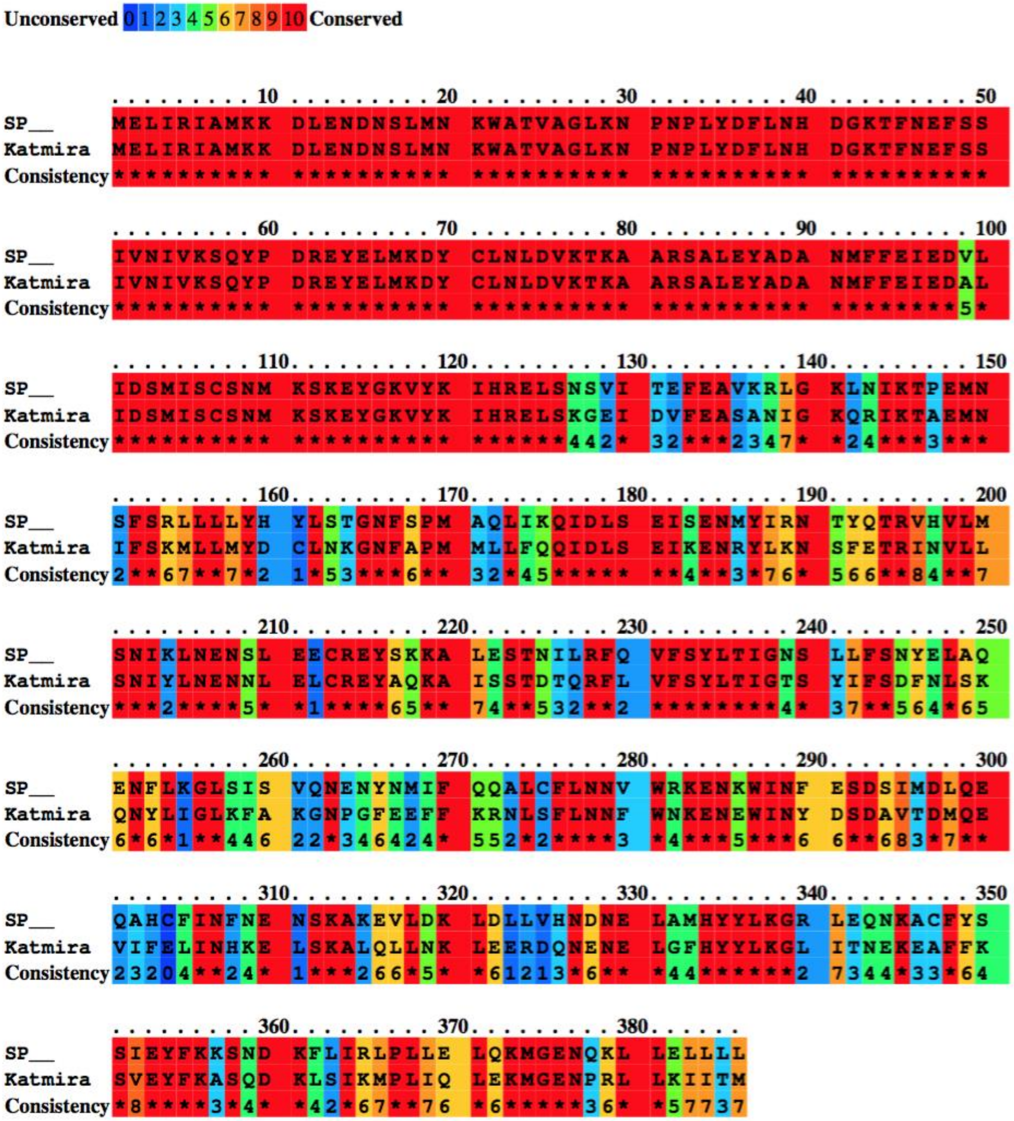

Figure S6. Homology analysis of $AimR_{SP\beta}$ and $AimR_{KATMIRA1933}$, Related to STAR Methods. AimR sequences from SPβ and KATMIRA1933 were obtained from BLAST. The superposition analysis was made using the PRALINE program. Residues conservancy is depicted by blue to red colours.

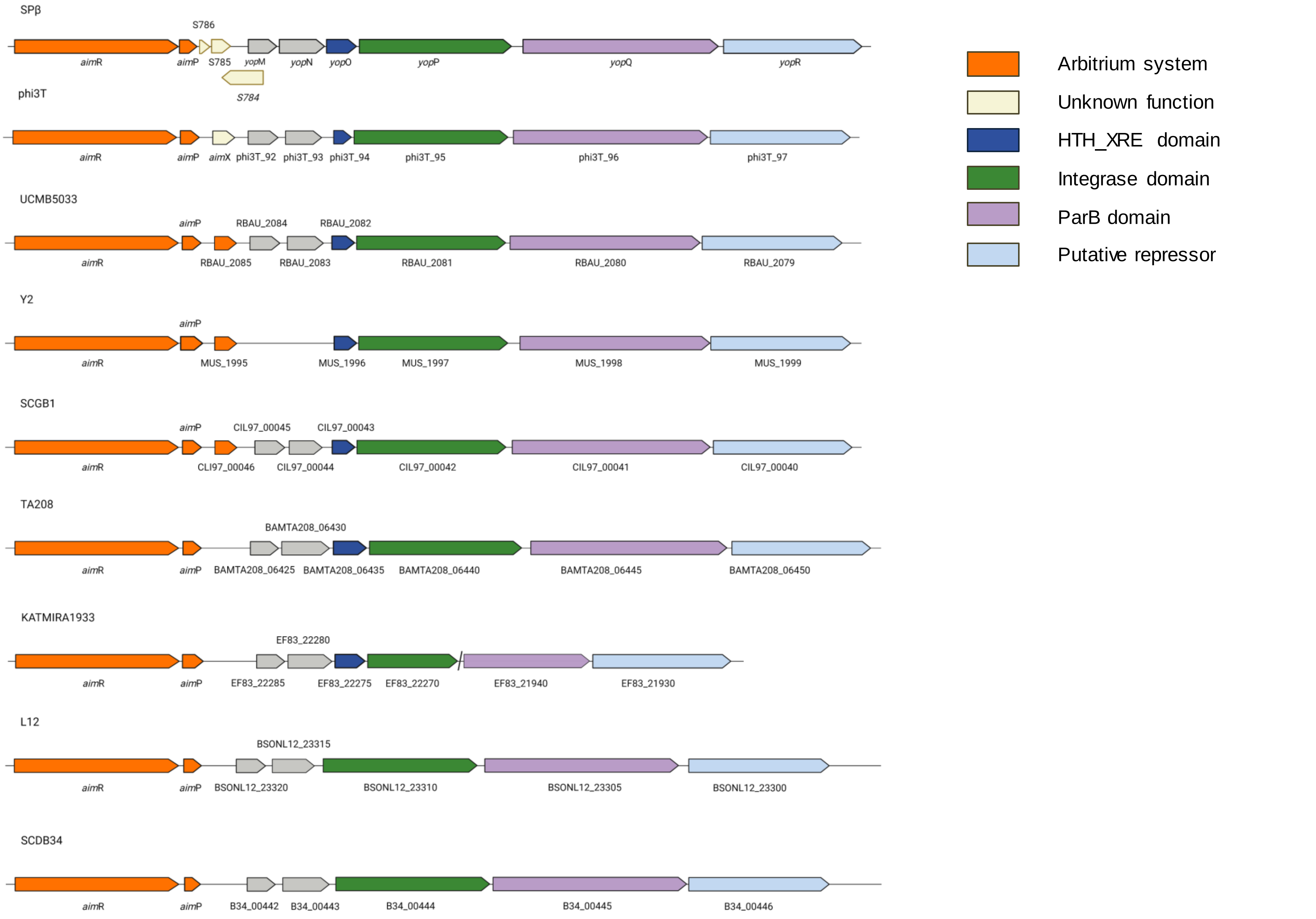

Figure S7. Schematic representation of the SPβ-like phages arbitrium and operon genetic layout, Related to Figure 3. Diagram shows the genetic organisation of the arbitrium genes, *aim*R and *aim*P, followed by the operon directly downstream. Colours denote putative functions according to BLAST results; orange: arbitrium genes, grey: unknown function, navy blue: HTH_XRE domain, green: integrase domain, purple: ParB domain, light blue: putative repressor. Rotated black line indicates the end/beginning of the contigs containing the genes described for Katmira1933. Created with BioRender.com

Strain	Gene	Mutation
JP20762	*yop*N	L90S
JP20766	*yop*N	L46P
	*yop*Q	T156T
JP20769	*yop*N	I51* Deletion produces frameshift and stop codon
Lytic phage 1	*yop*R	L140* Deletion produces frameshift and stop codon
Lytic phage 2	*yop*R	L49* Deletion produces frameshift and stop codon

Table S1. Mutations identified in evolved SPβ Δ*aim*R phages, Related to Figure 3 and Figure 4.

Phage/lysogen	AimR	AimP	AimP sequence	Operon genes accession numbers					
				Gene 1	Gene 2	Gene 3	Gene 4	Gene 5	Gene 6
SPβ	GenBank: NP_389968	GenBank: NP_389967	GMPRGA	GenBank: NP_389966	GenBank: NP_389965	GenBank: NP_389964	GenBank: NP_389963	GenBank: NP_389962	GenBank: NP_389961
phi3T	GenBank: APD21232	GenBank: APD21233	SAIRGA	GenBank: APD21235	GenBank: APD21236	GenBank: APD21237	GenBank: APD21238	GenBank: APD21239	GenBank: APD21240
Bacillus amyloliquefaciens UCMB5033	GenBank: CDG30054	*NA	SPSRGA	GenBank: CDG30052	GenBank: CDG30051	GenBank: CDG30050	GenBank: CDG30049	GenBank: CDG30048	GenBank: CDG30047
Bacillus velezensis strain SCGB 1	GenBank: ATC49385	GenBank: ATC49384	SIIRGA	GenBank: ATC49382	GenBank: ATC49381	GenBank: ATC49380	GenBank: ATC49379	GenBank: ATC49378	GenBank: ATC49377
Bacillus amyloliquefaciens TA208	GenBank: AEB23458	GenBank: AEB23459	GVVRGA	GenBank: AEB23460	GenBank: AEB23461	GenBank: AEB23462	GenBank: AEB23463	GenBank: AEB23464	GenBank: AEB23465
Bacillus atrophaeus BA59	GenBank: ATO28982	GenBank: ATO28981	GMPRGA	GenBank: ATO28980	*NA	GenBank: ATO28979	*NA	GenBank: ATO28978	GenBank: ATO28977
Bacillus subtilis KATMIRA1933	GenBank: WP_033885437	GenBank: WP_134819006	GIVRGA	GenBank: WP_033885435	GenBank: WP_009967507	GenBank: WP_019712296	GenBank: WP_033885434	GenBank: NP_389962.1	GenBank: WP_003231032
Bacillus sonorensis L12	GenBank: WP_051056713	GenBank: WP_141231111	GFPRGA	GenBank: WP_006640569	GenBank: WP_006640568	*NA	GenBank: WP_006640567	GenBank: WP_006640566	GenBank: WP_006640565
Bacillus licheniformis strain SCDB 34	GenBank: ARC67883	GenBank: ARC67884	GFTVGA	GenBank: ARC67885	GenBank: ARC67886	*NA	GenBank: ARC67887	GenBank: ARC67888	GenBank: ARC67889

*NA: Not annotated

Table S2. Genetic composition of the arbitrium-operon region in the different SPβ-like phage families, Related to Figure 3.

Strains	Genotype or description	Reference or source
Bacillus subtilis		
168 (1A700)	*trpC2*	S1
Δ6 (1A1299)	*trpC2*; ΔSPβ ; subclacin 168-sensitive; Δ*skin*; ΔPBSX; Δprophage 1; Δ*pks*::Cm; Δprophage 3; Cm^r	S2
IL26	phi3T	S3
BKK20860	*trpC2* Δ*aim*R::*kan*	S4
BKE20860	*trpC2* Δ*aim*R::*erm*	S4
BKE20850	*trpC2* Δ*aim*P::*erm*	S4
BKE20830	*trpC2* Δ*yop*N::*erm*	S4
BKE20790	*trpC2* Δ*yop*R::*erm*	S4
JP22770	*trpC2 SP*β Δ*aim*R	This study
JP22771	*trpC2 SP*β Δ*aim*P	This study
JP22776	*trpC2 SPβ ΔaimR*; *amy*E::P_{spank}	This study
JP22777	trpC2 SPβ ΔaimR; *amy*E::P_{spank}*aim*$R_{SPβ}$	This study
JP19877	Δ6 lysogenic SPβ	This study
JP19936	Δ6 lysogenic SPβ Δ*aim*R	This study
JP20866	Δ6 lysogenic SPβ *yok*I::*kan*	This study
JP22949	Δ6 lysogenic SPβ *yok*I::*kan* Δ*aim*R	This study
JP21702	Δ6 lysogenic SPβ *yok*I::*kan* Δ*aim*P	This study
JP22950	Δ6 lysogenic SPβ *yok*I::kan Δ*aim*R; *amy*E::P_{spank}	This study
JP22951	Δ6 lysogenic SPβ *yok*I::kan Δ*aim*R; *amy*E::P_{spank}*aim*$R_{SPβ}$	This study
JP21854	Δ6 lysogenic phi3T	This study
JP21870	Δ6 lysogenic phi3T *phi3T_5*::*kan*	This study
JP22453	Δ6 lysogenic phi3T *phi3T_5*::*kan* Δ*aim*R	This study
JP22454	Δ6 lysogenic phi3T *phi3T_5*::*kan* Δ*aim*P	This study
JP22518	Δ6 lysogenic phi3T *phi3T_5*::*kan* Δ*aim*R; *amy*E::P_{spank}	This study
JP22519	Δ6 lysogenic phi3T *phi3T_5*::*kan* Δ*aim*R; *amy*E::P_{spank}*aim*$R_{SPβ}$	This study
JP20762	Δ6 lysogenic SPβ Δ*aim*R; *yop*N L90S	This study
JP20766	Δ6 lysogenic SPβ *yok*I::*kan* Δ*aim*R; *yop*N L46P; *yop*Q T156T	This study
JP20769	Δ6 lysogenic SPβ *yok*I::*kan* Δ*aim*R; *yop*N A49*	This study
JP22952	Δ6 lysogenic SPβ Δ*yop*N	This study
JP22953	Δ6 lysogenic SPβ Δ*aim*R Δ*yop*N	This study
JP21752	Δ6 lysogenic SPβ *yop*R::*erm*	This study
JP22339	Δ6 lysogenic SPβ *yop*R::*erm; amy*E::P_{spank}*yop*$R_{SPβ}$	This study
JP19679	Δ6 *amy*E::P_{spank}	This study
JP19944	Δ6 *amy*E::P_{spank}*aim*$R_{SPβ}$	This study
JP22515	Δ6 *amy*E::P_{spank}*aim*R_{3T}	This study
JP21941	Δ6 *amy*E::P_{spank}*yop*$R_{SPβ}$	This study
JP19883	Δ6 lysogenic SPβ; *amy*E::P_{spank}	This study

Table S3. Bacterial strains, Related to STAR Methods.

Plasmid	Description	Reference or source
pDR244	*B. subtilis* thermosensitive vector containing Cre recombinase that allows excision of DNA fragments flanked by *lox*P sites	S4
pDR110	*B. subtilis amy*E integration vector containing IPTG-inducible P_{spank} promoter	S5
pJP2340	*aim*$R_{SP\beta}$ gene cloned in integration vector pDR110	This study
pJP2801	*aim*R_{3T} gene cloned in integration vector pDR111	This study
pJP2800	*yop*$R_{SP\beta}$ gene cloned in integration vector pDR110	This study

Table S4. Plasmids used in this study, Related to STAR Methods.

Mutants		Oligonucleotides	Sequence (5'-3')
kan marker without *lox*P		KanR-5m	TTTGATTTTTAATGGATAATGTGATATAATC
		KanR-6c	TCTAGGTACTAAAACAATTCATCC
erm marker with *lox*P		ErmR-1m	CAGGCGAGAAAGGAGAGAGAACGCAAGGA GAGGC ACGCGAGG GAGG AAA GGC AGGATACCGTTCGTATAGCATACATTATACGAAGTTATGAATTC
		ErmR-2c	GAGGCTCCTGTCACTGCTTCGCTCTGCTTCGGTGTCGTCGCCGTATCTGTGCTC TCTCTACCGTTCGTATAATGTATGCTATACGAAGTTATCTCGAG
SPβ *yok*I::*kan*	Forward Flanking	yokI-5pL	ATCCTCCATTGCTTTAGTCAGTATG
		yokI-1_R	GATTATATCACATTATCCATTAAAAATCAAACCATTTCATTCTCCTTTCAAGCC
	Reverse Flanking	yokI-4_F	GGATGAATTGTTTTAGTACCTAGAAACTTTAGAAAGTAGGTGCG
		yokI-3pR	ACTGAAGACAAACTCCTCAAACG
phi3T *yok*I::*kan*	Forward Flanking	phi3T-1m	GCAATGTTTCCTGAACAGATTTGC
		phi3T-2c	GATTATATCACATTATCCATTAAAAATCAAAATCATTCTCCTTCCATTCTTACTC
	Reverse Flanking	phi3T-3m	GGATGAATTGTTTTAGTACCTAGACACAGGCCGAAGCTGAAGATTGG
		phi3T-4c	CTTGCCTACAACCTCCGCTTC
SPβ *aim*R::erm		AimR-SPB-24mB	CGCGGATCCTATACAATGGCGCTGAGATCC
		AimR-SPB-14cS	ACGCGTCGACCACAAAATGTATTAGGGATCTAAAATGCGG
SPβ *aim*P::erm		AimP-SPB-1mB	CGCGGATCCGACAAAGGCAGCAAGAAGTGC
		AimP-SPB-4c	ATTGTGATGCCACGTTTGACC
SPβ *yop*N::erm		Spbeta_5_S_F	CTGCAGGTCGACACCTGAAATGAATTCTTTCTCAAG
		YopMNO_R	GCCTTTCACCTCATGTCATGTTGC
SPβ *yop*R::erm		YopR_F	CTTCACAGAAACGGATATGAGAG
		YopR_R	CTCTCCCTTGAAACAAAGTAGG
phi3T *aim*R::*erm*	Forward Flanking	AimR-phi3T-1m	CGAATCGTGGAGAAACTTTGCAAATG
		AimR-phi3T-2c	GTTCTCTCTCCTTTCTCGCCTGCCTGCTTTAATTTCAATTGTCTCC
	Reverse Flanking	AimR-phi3T-3m	GCGAAGCAGTGACAGGAGCCTCGGTTTGACAAATTTGAAAGGA GGTG
		AimR-phi3T-4c	CAAGACAATCATATGCTTTTTCCAG
phi3T *aim*P::*erm*	Forward Flanking	AimR-phi3T-5m	GTTGCATTTGGCCAATTATGC
		AimR-phi3T-11c	GCCAATAGTTAAGTAGCTGAAAACCTGAAACGCCAGGATATTTGTACTTTCCAA TGCC
	Reverse Flanking	AimR-phi3T-11m	GGCTTTGTGCTTCTTAAATAATGTATGGCGCGCCGCCGCCAAGTGGATTAATTT TGAATCTGATTCAATTATGG
		AimR-phi3T-4c	CAAGACAATCATATGCTTTTTCCAG
**Plasmids**		**Oligonucleotides**	**Sequence (5'-3')**
pJP2340		AimR-SPBeta-1mH	CCCAAGCTTGACTCGTAATGTGATCTATAG
		AimR-SPBeta-2cS	ACGCGTCGACCATTGTCTCACCTCCTTTAAAGTAAAAG
pJP2750		AimR-phi3T-9mS	ACGCGTCGACCTTGAAATTCTGACAACTATGAGG
		AimR-phi3T-10cSphI	ACATGCATGCCCTCCTTTCAAATTTGTCAAACC
pJP2800		YopR_2F	ACGCGTCGACAGGTGTAGTAGACAAGAATGG
		YopR_2R	ACATGCATGCCCATTTAACCAAAATAGTCAAATGGATTTC
**Southern Blot**		**Oligonucleotides**	**Sequence (5'-3')**
SPβ probe		SPBeta-1m	GATAGGCTTACCGAGGTCTTC
		SPBeta-2c	CTAATGGACGGCTGGAGAGGC

Table S5. Primers used in this study, Related to STAR Methods.

Supplemental References:

S1. Burkholder, P. R., and N. H. Giles. (1947). Induced biochemical mutations in *Bacillus subtilis*. Amer. J. Bot. 34:345.

S2. Westers H, Dorenbos R, van Dijl JM, Kabel J, Flanagan T, Devine KM, *et al*. (2003). Genome engineering reveals large dispensable regions in *Bacillus subtilis*. Mol Biol Evol. *20,* 2076–90.

S3. Dean, D. H., J. C. Orrego, K. W. Hutchinson, and H. O. Halvorson. (1976). New temperate bacteriophage for *Bacillus subtilis*, r11. J. Virol. *20,* 509-519.

S4. Koo BM, Kritikos G, Farelli JD, Todor H, Tong K, Kimsey H, Wapinski I, Galardini M, Cabal A, Peters JM *et al*. (2017). Construction and Analysis of Two Genome-Scale Deletion Libraries for *Bacillus subtilis*. Cell Syst. *4,* 291-305.

S5. Carniol K, Ben-Yehuda S, King N, Losick R. (2005). Genetic dissection of the sporulation protein SpoIIE and its role in asymmetric division in *Bacillus subtilis*. J Bacteriol. *187*, 3511–20.
